# A review on gasification and pyrolysis of waste plastics

**DOI:** 10.3389/fchem.2022.960894

**Published:** 2023-02-03

**Authors:** Hamad Hussain Shah, Muhammad Amin, Amjad Iqbal, Irfan Nadeem, Mitjan Kalin, Arsalan Muhammad Soomar, Ahmed M. Galal

**Affiliations:** ^1^ Department of Engineering, University of Sannio, Benevento, Italy; ^2^ Department of Energy Systems Engineering, Seoul National University, Seoul, Republic of Korea; ^3^ Department of Materials Technologies, Faculty of Materials Engineering, Silesian University of Technology, Gliwice, Poland; ^4^ CEMMPRE - Centre for Mechanical Engineering Materials and Processes, Department of Mechanical Engineering, Rua Luís Reis Santos, Coimbra, Portugal; ^5^ Laboratory for Tribology and Interface Nanotechnology, Faculty of Mechanical Engineering, University of Ljubljana, Ljubljana, Slovenia; ^6^ Faculty of Electrical and Control Engineering, Gdańsk University of Technology, Gdańsk, Poland; ^7^ Mechanical Engineering Department, College of Engineering, Prince Sattam Bin Abdulaziz University, Wadi ad-Dawasir, Saudi Arabia; ^8^ Production Engineering and Mechanical Design Department, Faculty of Engineering, Mansoura University, Mansoura, Egypt

**Keywords:** gasification, pyrolysis, plastic waste, valorization, chemistry

## Abstract

Gasification and pyrolysis are thermal processes for converting carbonaceous substances into tar, ash, coke, char, and gas. Pyrolysis produces products such as char, tar, and gas, while gasification transforms carbon-containing products (e.g., the products from pyrolysis) into a primarily gaseous product. The composition of the products and their relative quantities are highly dependent on the configuration of the overall process and on the input fuel. Although in gasification, pyrolysis processes also occur in many cases (yet prior to the gasification processes), gasification is a common description for the overall technology. Pyrolysis, on the other hand, can be used without going through the gasification process. The current study evaluates the most common waste plastics valorization routes for producing gaseous and liquid products, as well as the key process specifications that affected the end final products. The reactor type, temperatures, residence time, pressure, the fluidizing gas type, the flow rate, and catalysts were all investigated in this study. Pyrolysis and waste gasification, on the other hand, are expected to become more common in the future. One explanation for this is that public opinion on the incineration of waste in some countries is a main impediment to the development of new incineration capacity. However, an exceptional capability of gasification and pyrolysis over incineration to conserve waste chemical energy is also essential.

## 1 Introduction

Plastics are adaptable, flexible, and lightweight, allowing them to be used in a wide variety of applications. In recent years, the political agenda has focused on the economic, environmental, and social influences of plastics, with an emphasis on sustainable manufacturing and the decoupling of negative ecological outcomes from waste generation. Waste plastics disposal has become a significant global environmental issue. Around 55 million tons of postconsumer plastic waste are produced annually in the United States, Japan, and Europe (Sun et al., 2021). Previously, these waste products were discarded in landfills, which was an unsustainable and environmentally unsound practice. Furthermore, the number of landfill sites and their capabilities are steadily declining, and landfill regulation is becoming more stringent in most countries. Recycling is being considered as another option for managing plastic waste in order to reduce its disposal in landfills. Because of the restrictions on water pollution and insufficient separation prior to recycling, which is labor intensive, recycling plastic has proved difficult and expensive (Jaafar et al., 2022). Since plastics come in a variety of colors, resin compounds, and transparencies, separation is required. Plastics that are pigmented or dyed typically have a lower market value. Manufacturers choose clear transparent plastics because they can be colored and turned into new goods, giving them more flexibility (Thompson, 2022). Recycling plastic has become difficult in recent years due to the strict requirements for obtaining high-value products.

**FIGURE 1:**
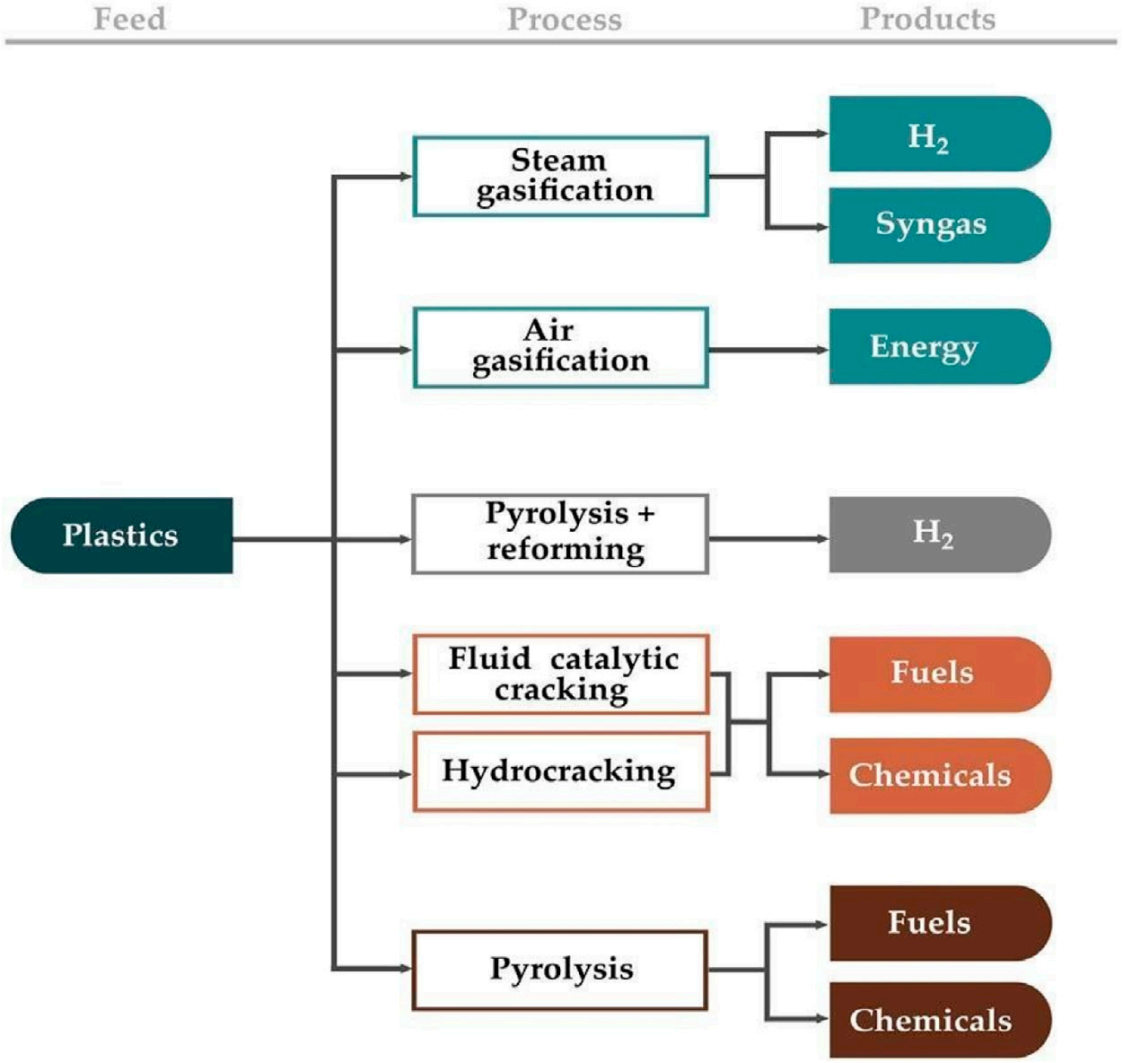
Primary chemical routes for plastic waste valorization.

The disposal of plastic waste presents a significant problem that must be tackled immediately. As a result, plastics’ low degradability poses significant ecological issues, particularly in marine environments (de Sousa, 2021). Furthermore, insufficient waste plastics management contributes to environmental concerns due to the depletion of essential and limited resources obtained from petroleum. As a result, in recent years, public policies aimed at strengthening waste plastics management have been promoted. In fact, in Europe over the last decade, the quantity of plastic waste disposed of in landfills has decreased by 38% while the fraction of waste plastics used for energy valorization and recycling has increased by 46% and 64%, respectively (Plastics, 2016). Although the situation with waste plastics management in developed countries is slowly improving, it is still far from satisfactory, and in developed countries, plastics management is obviously less promising. Different methods, such as reuse, recycling, energy recovery, and waste minimization are being considered with the goal of minimizing the volume of waste that is disposed of in landfills. However, neither minimization nor reuse has been extensively utilized in the case of waste plastics (Aguado et al., 2008). Combustion is a viable valorization route due to the high calorific value of plastics, but it is hampered by the emissions generated (Thimoteo et al., 2022). Chemical recycling routes have been the best chance of being implemented on a wide scale because these permit the formation of syngas/hydrogen, chemicals, and fuels from plastic waste. Figure 1 depicts the major chemical valorization pathways for waste plastics. Pyrolysis of waste plastics is widely recognized as the most efficient method for producing chemicals and fuels from plastic waste (Aguado et al., 2008), (Al-Salem et al., 2009; Al-Salem et al., 2010; Butler et al., 2011; Wong et al., 2015; Anuar Sharuddin et al., 2016; Kunwar et al., 2016; Ma et al., 2016; Yu et al., 2016; Lopez et al., 2017).

The solid waste incineration is an attractive technology for thermal energy generation and reducing the volume of landfill waste. However, municipal waste incineration involves climate-relevant emissions (CO_2_, SOx, NOx, and N_2_O). One tonne incineration of municipal waste generates about 0.7–1.7 tonnes of CO_2_, thus making significant greenhouse effect contribution. When compared to other conventional plastic recycling techniques (such as gasification and pyrolysis), the energy produced by incineration has significantly high emissions of greenhouse gases (340 g CO_2_ eq per kWh). Therefore, waste incineration is not an environmentally friendly alterative due to subsequent greenhouse gas emissions.

Various plastic pyrolysis processes have been developed for the selective processing of waxes (Berrueco et al., 2002; Arabiourrutia et al., 2012a; Yansaneh and Zein, 2022), light olefins (Milne et al., 1999; Mastral et al., 2006a; Hernandez et al., 2007; Elordi et al., 2011; Artetxe et al., 2013a), and monomers (Achilias et al., 2007; Mo et al., 2014). Furthermore, in recent years, plastic waste and biomass co-pyrolysis have gained a lot of attention (Xue et al., 2015; Zhang et al., 2016a). Despite the growing interest in plastic waste pyrolysis, it is still in the developmental stages of implementation (Butler et al., 2011). Waste plastics and their derivatives, such as pyrolysis wax oil products, can also be fed into traditional refinery units to produce fuels (Arandes et al., 1997; Lopez et al., 2017; Lovás et al., 2017; Palos et al., 2022a).

Numerous studies have been conducted on the potential of different plastic types for gasification and pyrolysis procedures to produce gas and liquid products. It is important to note that the setup parameters have a significant impact on product quality and yield. Therefore, this review concentrates on the various plastic gasification and pyrolysis processes that have been investigated along with the key factors that affect these processes and those that require attention in order to maximize the production of gas and liquid oil and improve the quality of the final product. The primary parameters include pressure, residence time, the reactor type, temperature, the use of various catalysts, and the type and flow rate of the fluidizing gas. The obtained results from various valorization methodologies have been compared, and their potential values have been discussed critically. Furthermore, this study also presents some important discussion concerning product yield optimization.

## 2 Gasification

By partial oxidation with a gasification agent, gasification refers to the chemical and thermal conversion of carbon-based materials into a primarily gaseous output (usually air, oxygen, or steam). If gasification is preceded by pyrolysis, the pyrolysis outputs (gas, tar, and char) can be improved further by partial oxidation of the more complex hydrocarbons, particularly those found in the char and tar.

Temperature range from 800 to 1,100°C when using air as an oxidant, and up to 1,500°C when using oxygen. While most gasification processes are exothermal, that is, they generate heat, some of the associated reactions are endothermal and require heat, which could be provided by steam as the gasification agent. In general, the products of gasification are

**Solid:** non-volatile metals and other inorganic elements are found in ashes. Solids may account for 30–50% of the input weight.

**Liquid:** smaller amounts of oil and tar, about 10–20% by weight of the input, are used in some conditions.

**Gas:** same as pyrolysis gas but with higher CO_2_ fractions. The heating value varies depending on the gasification agent, but it is usually 3–12 MJ/Nm^3^ with oxygen as the gasification agent. By weight of the supply, the gas yield can range from 30 to 60% (Belgiorno et al., 2003; Hu et al., 2021; Tezer et al., 2022).

Like pyrolysis products, gasification products are strongly influenced by the temperature, waste input, and overall process framework. The waste input, in particular, is often underrepresented in the literature, and the waste is frequently composed of distinct industrial segments instead of mixed MSW. The heating value for the gas output can therefore be considered as the upper limit for MSW. Char and tar formed by pyrolysis reactions are further converted to CO_2_, CO, CH_4_, and H_2_ by heating to higher temperatures than pyrolysis and adding a gasification agent. The gasification agent used has a considerable impact on the processed gas composition, and “dilution” from the gasification agent has a substantial impact on the gas heating value, again contingent on the agent (medium) used. For example, air gasification is less expensive than using pure oxygen as a gasification agent but produces a gas that contains up to 60% nitrogen (Tezer et al., 2022).

## 3 Chemical reactors for gasification of plastic waste

Plastic waste gasification processes are exactly the same as those used to gasify other feedstocks such as coal and biomass. However, the unique properties of plastic wastes, particularly their high volatility and high thermal resistivity; sticky, viscous, and adhesive nature; and exceptional tar production, obstruct their processing in traditional gasification technologies and pose a significant challenge for process realization. As a result, an adequate gasifier design for plastic handling must incorporate the following characteristics: it should 1) be capable of providing high rates of heat transfer aiming to facilitate rapid depolymerization of plastic waste, 2) evade operative issues caused by the sticky and adhesive behavior of plastics by maintaining a tight control over the operating parameters and conditions, 3) have adequate residence time dispensation to favor the cracking of tar and enable the use of primary (fundamental) catalyst *in situ* while maintaining virtuous contact with the catalyst.

Traditional waste gasification systems are fixed bed, entrained flow, downdraft, updraft, fluidized bed, plasma reactor, and rotary kiln (Heidenreich and Foscolo, 2015; Ahmad et al., 2016; Mahinpey and Gomez, 2016; Molino et al., 2016; Ud Din and Zainal, 2016; Sansaniwal et al., 2017). However, because of the complexities of waste plastics, some of these technologies have been limited in their application. Each gasification system is available in a number of basic configurations, each with benefits for a specific product or feedstock applications. Each system type’s basic design revolves around the reaction chamber with feedstock insertion, but each has a unique air entry, heating mechanism, and syngas removal area.

### 3.1 Spouted conical bed gasifier

**FIGURE 2:**
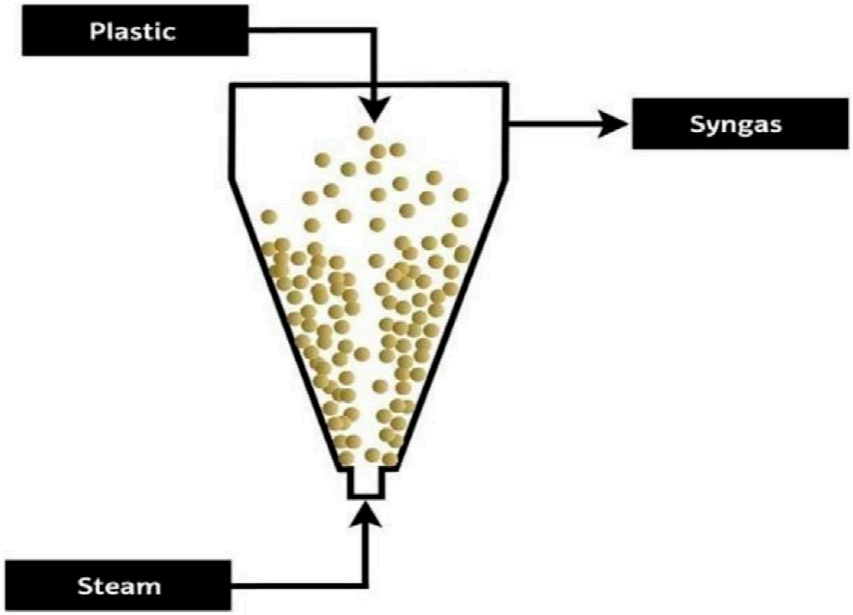
Spouted conical bed gasifier.

Conical spouted reactors are a substitute for heterogeneous fluidized beds (FBRs) for waste valorization processes due to their unique characteristics. As a result, these reactors have high mass and heat transfer rates, appropriate fluidization, and excellent solid mixing (Makibar et al., 2011). Furthermore, their dynamic solid cyclic circulation eliminates agglomeration and de-fluidization issues and facilitates the manipulation of irregular and discrete particulates, particles with a wide distribution size, and adhesive substances. In gasification processes, the primary drawbacks are the volatiles’ short residence (stay) time, which impedes the cracking tar reactions (Erkiaga et al., 2014). In bench-scale units, this technology is extensively applied in the pyrolysis of various solid wastes (Lopez et al., 2009; Lopez et al., 2010; Amutio et al., 2012; Artetxe et al., 2013a; Alvarez et al., 2015). Furthermore, the biomass pyrolysis process has been effectively generalized up to 25 kg/h (Fernandez-Akarregi et al., 2013; Makibar et al., 2015). The first time the spouted beds were used in the gasification processes, coal was used as the feedstock (Foong et al., 1981; Teo and Watkinson, 1986; Sueaquan et al., 1995; Fernandez et al., 2022). Gasification of different feedstocks (raw materials) has recently been added to this technology, such as waste plastics and biomass (Erkiaga et al., 2013a; Erkiaga et al., 2013b; Bernocco et al., 2013; Erkiaga et al., 2014; Lopez et al., 2015a; McCullough et al., 2015). To decrease the content of tar and improve the efficiency of the process in the gaseous product, various primary (fundamental) catalysts have been investigated *in situ* (Erkiaga et al., 2013a; Erkiaga et al., 2013b), or in a second reactor, secondary catalysts have been utilized (Lopez et al., 2015b). Figure 2 depicts a spouted conical bed gasifier design.

### 3.2 Fixed (packed) bed reactor

Packed bed reactors are used in the gasification of plastic because of the ease in their operation and design, and their low investment cost, with the key problem being scaling up, limited gas–solid contact, continuous operation, and a low heat transfer rate. There are many different designs of fixed-bed reactors, but they all have one thing in common: they are used in small-scale units (Ahmed and Gupta, 2009; Wu and Williams, 2010a; Wu and Williams, 2010b; Wu and Williams, 2010c; Friengfung et al., 2014; Parparita et al., 2015; Baloch et al., 2016). Usually, plastic waste gasification (Ponzio et al., 2006; HeMXiao et al., 2009a; Wang et al., 2012; Lee et al., 2014; Ongen, 2016) or their coprocessing with biomass and coal (Straka and Bicáková, 2014a; Akkache et al., 2016; Singh et al., 2022) in fixed-bed reactors has received little attention. Ahmed and Gupta (2009) used a laboratory-scale fixed-bed (packed) reactor operating in the batch mode for steam co-gasification of polystyrene and plastic–wood samples (Singh et al., 2022). Moreover, experiments were performed in a bench-scale fixed-bed reactor designed by HeMXiao et al. (2009b) at a plastic continuous feed rate of 0.3 kg/h, and the impact of utilizing reforming *in situ* Ni/Al_2_O_3_ catalyst was investigated. Li et al. (2012) also developed a similar continuous-mode experimental setup for MSW steam gasification. Lee et al.( 2014) conducted their research in a semi-batch laboratory-scale reactor with a steam (condensation) atmosphere. Guo et al. (2015, 2016) investigated polyurethane air gasification by utilizing various *in situ* catalysts in a laboratory-scale fixed-bed (packed) reactor.

### 3.3 Fluidized bed reactors

**FIGURE 3:**
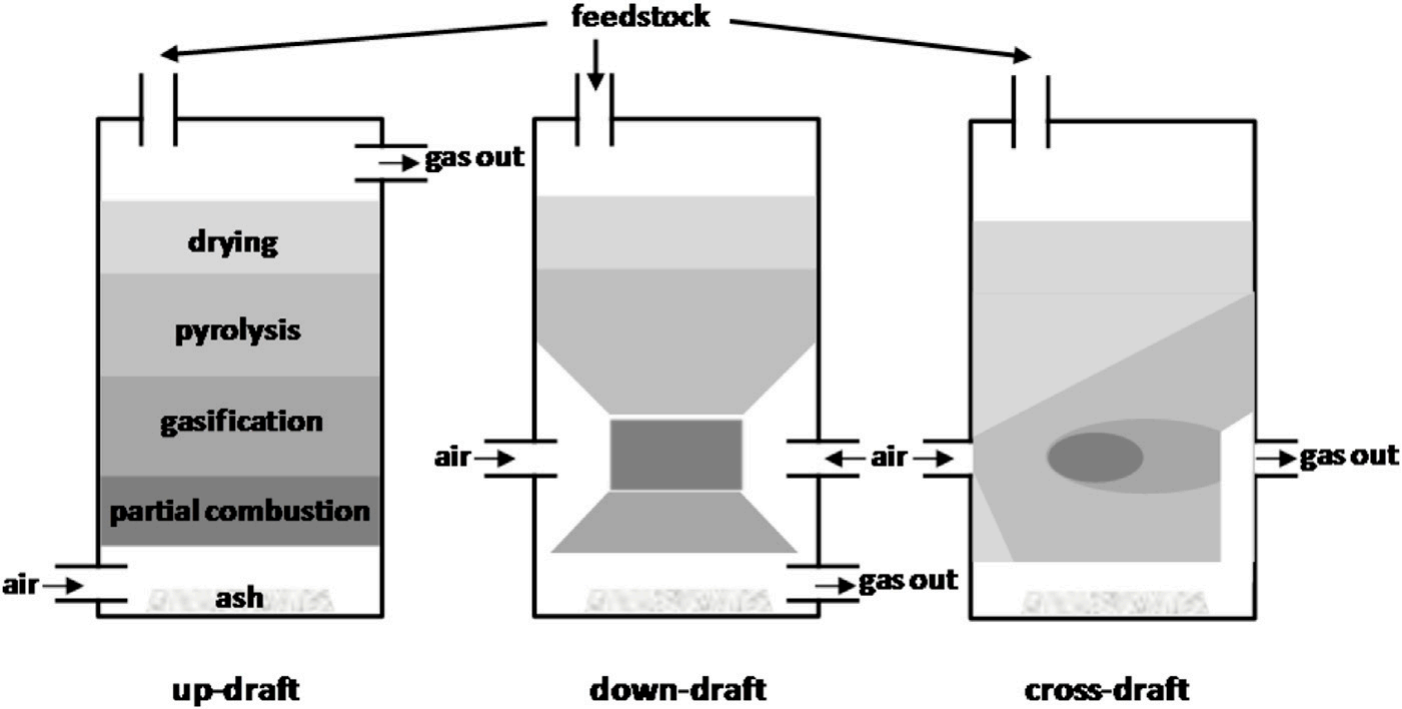
Gasification system types.

In gasification processes, two classes of fluidized bed reactors have traditionally been utilized: circulating fluidized beds (CFBs) and bubbling fluidized beds (BFBs) (Mahinpey and Gomez, 2016; Molino et al., 2016). Despite the intriguing characteristics of CFBs for the gasification of plastic waste operations, particularly the ability to achieve low tar and high conversion yields (McKendry, 2002), plastic gasification research has been limited to BFBs. The primary benefits of BFBs are their excellent gas–solid contact, high mass and heat transfer rates, good temperature control and flexibility, and good solid-mixing regime. Their primary drawbacks are their limitations in particle size both in feed and bed, high investment cost, unreacted material entrainment, and defluidization issues (Molino et al., 2016). These reactors run in a continuous mode and have a high scale and development degree, with various research being conducted in pilot plant scale units (Arena et al., 2010; Arena et al., 2011; Ruoppolo et al., 2012; Martínez-Lera et al., 2013a; Wilk and Hofbauer, 2013; Arena and Di Gregorio, 2014; Brachi et al., 2014; Narobe et al., 2014). In the co-gasification with coal and biomass or in plastic waste gasification, these are generally used with air as the gasifying medium (Sancho et al., 2008; Kim et al., 2011; Toledo et al., 2011; Ruoppolo et al., 2012; Cho et al., 2013a; Martínez-Lera et al., 2013b; Martínez-Lera et al., 2013c; Arena and Di Gregorio, 2014; Brachi et al., 2014). Despite the low gas heating value obtained, this approach offers functional benefits like lesser content of tar in the product gas and autothermal process (Gil et al., 1999; Devi et al., 2003). Mastellone and Arena (2008), Arena et al. (2009), and Arena et al. (2010) conducted research with continuous feed rates in a pilot plant up to 100 kg/h, while gasifiers have been employed by other researchers with feed rates of plastic ranging from 1 to 4 kg/h, running in a continuous mode (Xiao et al., 2007; Sancho et al., 2008; Toledo et al., 2011). Because steam gasification is considerably endothermic, it has high requirements of energy that are resolved in biomass gasification by utilizing dual fluidized bed (DFB) reactors, which combine a fast fluidized bed puffed with air with a steam-blown fluidized bed, where the residual char is burned (Goransson et al., 2011; Schneider et al., 2022). The research group led by Prof. Hofbauer used this operating methodology to gasify waste plastics in a pilot plant with a capacity of 15 kg/h (Martínez-Lera et al., 2013a; Narobe et al., 2014). However, as a result of the low yield of char and the problem in maintaining the heat balance between the combustion and gasification operations, issues may arise. Figure 3 depicts various types of gasifier schemes.

### 3.4 Plasma gasification reactors

**FIGURE 4:**
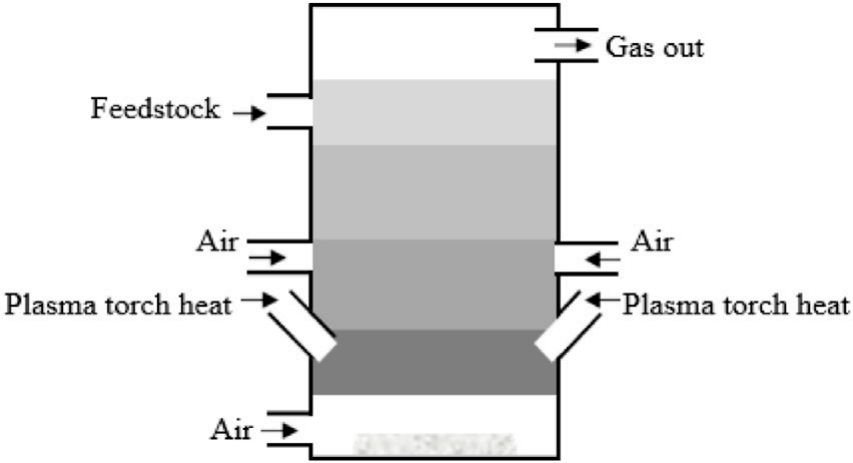
Plasma gasification (Oliveira et al. 2022).

The primarily use of plasma gasification is in industries where hazardous waste is disposed of at relatively high temperatures. The plasma torch in the gasifier (Figure 4) generates high temperatures (up to 10,000°F). There are two plasma gasification arrangements available depending on where the plasma torch is used in the gasification process. The first is plasma-assisted gasification, and the second is plasma-assisted gasification combined with traditional thermal gasification. This methodology has been utilized sparingly for the gasification of plastic waste, and studies have usually been conducted on a small scale (Tang and Huang, 2007; Rutberg et al., 2013a; Gibadullina et al., 2015; VishwajeetPawlak-Kruczek et al., 2022). However, the level of development achieved by Hlina et al. (2014) in their gasification unit, which works in a continuous mode with 11 kg/h plastic feed rate, is remarkable. Park et al. (2016) proposed combining continuous pyrolysis processes with 1.3 kg/h feed rate in a plasma reactor with gasification–pyrolysis (in-line) of volatiles.

## 4 Temperature and heating rate

**FIGURE 5:**
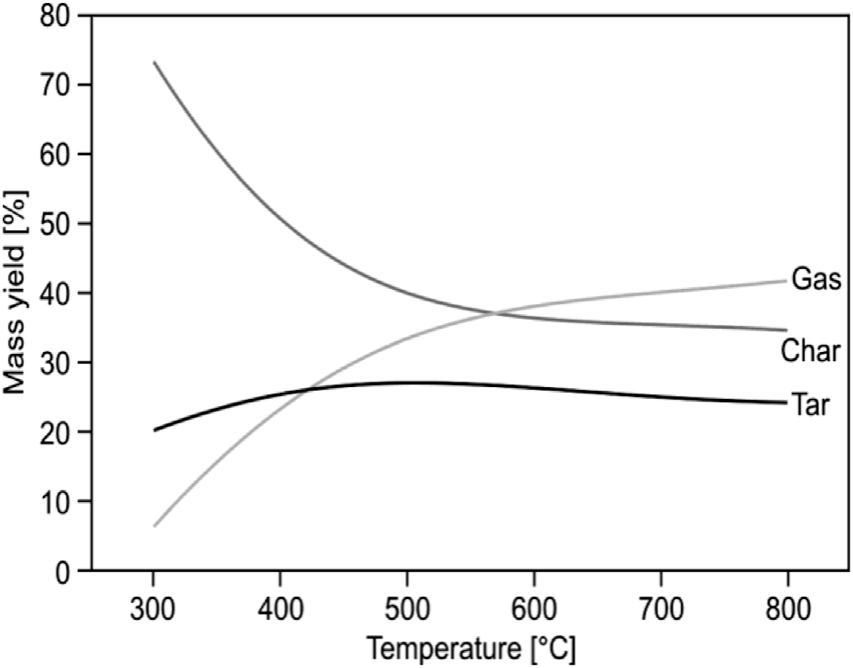
Reactor temperature (°C; RT) and mass yields (wt%, MY) correlation (Tezer et al., 2022).

The temperature reached in the reactor is critical because temperature changes affect the majority of the chemical reactions for waste conversion. Higher temperatures, in general, alleviate lower tar content and higher carbon conversion in the waste in the gas phase, but in the case of gasification, a lower heating value of the gas may also result. In pyrolysis, higher temperatures produce more gas, while lower temperatures produce more liquid. Figure 5 depicts a relationship between temperature and output products, demonstrating that temperature is a very important factor and that uniform distribution of temperature across the reactor is crucial.

Another factor that affects the outputs significantly is the heating rate (Hu et al., 2021). Char generation is increased by slow heating rates combined with relatively low final temperatures (e.g., slow heating at relatively low temperatures is required for the production of charcoal from wood). Mild heating rates up to mild temperatures give a more even weight distribution of pyrolysis outputs. High heating rates to high temperatures, possibly accompanied by rapid quenching, are commonly referred to as flash pyrolysis and can result primarily in a liquid product; however, the oils can be further broken down to enhance the gas output, if quenching is evading. Slow heating rates to high final temperatures typically result in a primarily gaseous product.

Some gasification processes use steam as the gasification agent and operate at high pressures (up to about 20 bar). High pressure favors the gas yield, though these processes may be circumscribed in their use with fuel as waste.

## 5 Gasification mechanism

**FIGURE 6:**
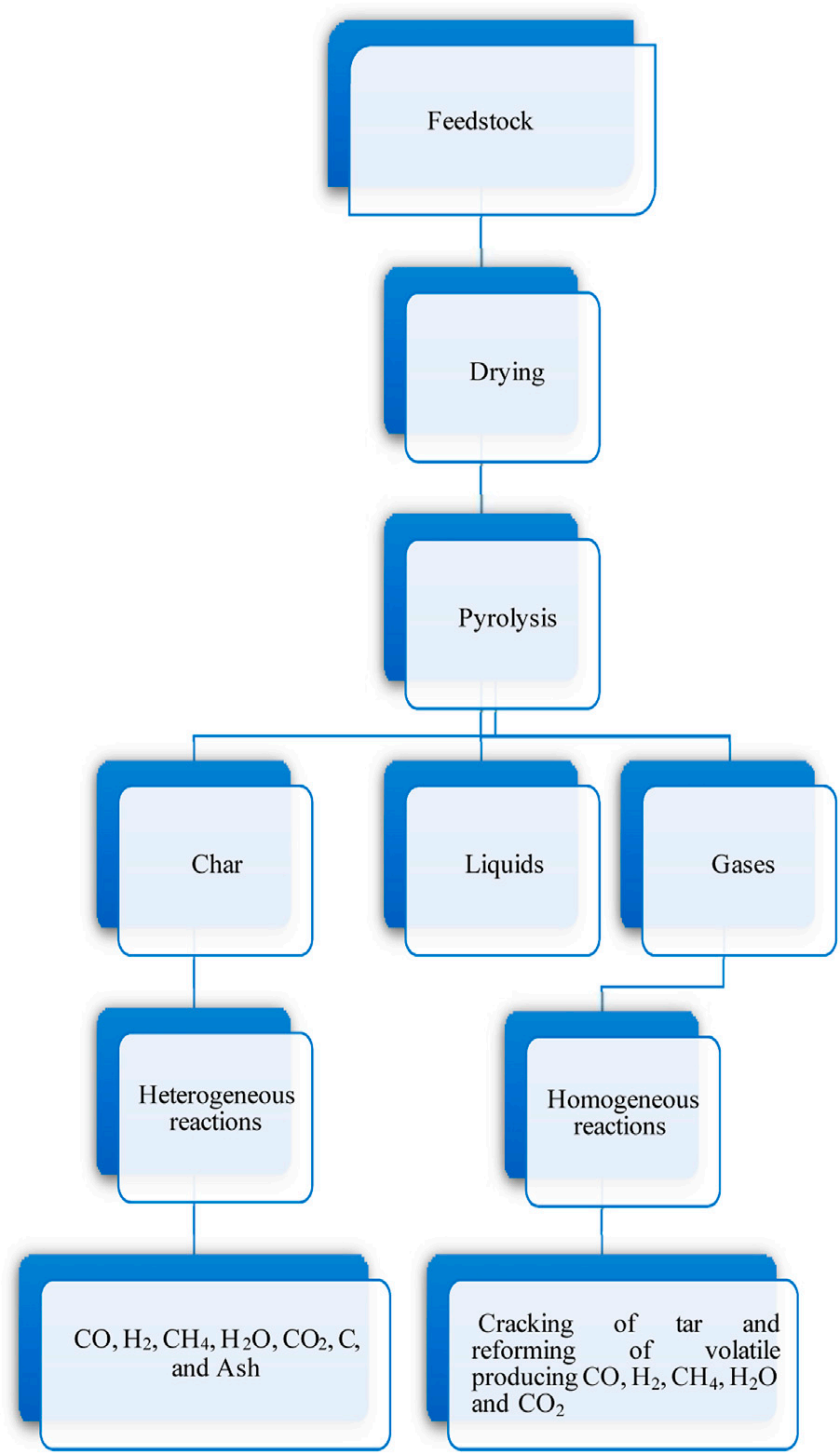
Potential gasification routes (Aguado et al., 2008).

The plastics gasification aims for the highest possible conversion to a syngas or gas product, with char and tar being the most unwanted derivatives. Gasification is a complex process and consists of many chemical reactions. Figure 6 depicts these steps. The importance of these steps in terms of kinetics and process performance is determined by the gasification conditions and feedstock properties. The main steps of gasification are

• Drying: around temperatures between 20 and 100°C, moisture is converted into steam. The feedstock is not decomposed, and no chemical reaction occurs at these temperatures. The predominant part of the gasification system is feedstock with a moisture content ranging from 10 to 20% for a high calorific value of produced gas.
• Pyrolysis: is devolatilization (thermal degradation), at temperatures between 150 and 700°C in the absence of oxygen, of the dry feedstock, liberating the volatile elements and a residue consisting of ash and char. The produced volatiles are a mixture of hydrogen, CO_2_, tar, CO, water vapor, and light hydrocarbons.
• Oxidation: in a gasification scenario, various oxidation chemical reactions occur, liberating the heat required for endothermic reactions. Carbon dioxide is produced due to the reaction between oxygen and char. Water is produced by oxidizing the hydrogen in the feedstock. Substoichiometric amounts of oxygen are present; partial oxidation of carbon may transpire, ensuing carbon monoxide production.
• Reduction: due to the consumption of oxygen in oxidation reactions, several chemical reactions, primarily endothermic ones occur in the absence of O_2_. CH_4_, CO, and H_2_ are the reduction reactions' main products.

The following is a list of the most important chemical reactions that take place during the gasification process:

$$Carbon\;reactions\;C+{CO}_{2}\;\to \;2CO+172\;MJ\;/\;kmol$$ (1)

$$Water-gas\;or\;steam\;C+H_{2}O\;\to \;CO+H_{2}+131\;MJ\;/\;kmol$$ (2)

$$Hydrogasification\;C+2H_{2}\;\to {CH}_{4}\;–\;74.8\;MJ\;/\;kmol$$ (3)

$$C+0.5O_{2}\;\to \;{CH}_{4}\;–\;111\;MJ\;/\;kmol$$ (4)

$$Oxidation\;reactions\;C+O_{2}\;\to \;{CO}_{2}\;–\;394\;MJ\;/\;kmol$$ (5)

$$CO+0.5O_{2}\;\to \;{CO}_{2}\;–\;284\;MJ\;/\;kmol$$ (6)

$${CH}_{4}+2O_{2}\;\to \;{CO}_{2}\;–\;803\;ML\;/\;kmol$$ (7)

$$H_{2}+0.5O_{2}\;\to \;H_{2}O\;–\;242\;MJ\;/\;kmol$$ (8)

Shift reaction

$$CO+H_{2}O\;\to \;{CO}_{2}+H_{2}O\;–\;41\;MJ\;/\;kmol$$ (9)

$$Methanation\;reaction\;2CO+2H_{2}\;\to \;{CH}_{4}+{CO}_{2}\;–\;247\;MJ\;/\;kmol$$ (10)

$$CO+3H_{2}\;\to \;{CH}_{4}+H_{2}O\;–\;206\;MJ\;/\;kmol$$ (11)

$${CO}_{2}+4H_{2}\;\to \;{CH}_{4}+2\;H_{2}O\;–\;165\;MJ\;/\;kmol$$ (12)

Steam reforming reaction

$${CH}_{4}+0.5O_{2}\;\to \;CO+2H_{2}\;–\;36\;MJ\;/\;kmol$$ (13)

$${CH}_{4}+H_{2}O\;\to \;CO+3H_{2}+206\;MJ\;/\;kmol$$ (14)

The gasification process, as far as can be determined, is globally endothermic, with the required heat obtained in one of the two ways: direct (autothermal) gasification occurs when heat is generated inside the reactor as a result of exothermic reactions, while indirect (allothermal) gasification occurs when the required heat is generated outside of the reactor (Milhé et al., 2013).

## 6 Processes involved in gasification of plastic waste

Valorization of waste plastics through gasification processes has been considered using a variety of schemes, with the goal of producing syngas of various compositions and potential applications. Research on the gasification of waste plastics is still in its early stages, and the number of studies is restricted. On the other hand, investigations on biomass and coal co-gasification have been conducted.

Due to process simplification, air gasification is the most widely used process as there are no external energy prerequisites. Moreover, as compared with steam gasification, tar content is typically lower in the gas products (Gil et al., 1999). As a result, this gas is primarily used in the production of energy (Arena et al., 2010; Arena, 2012). Steam gasification produces an H_2_-rich syngas with high ratios of H_2_/CO, which is more suitable for chemical synthesis applications than direct air gasification syngas (Erkiaga et al., 2013a). The main difficulty with this alternative is the amount of heat that must be introduced into the reactor in order to perpetrate the endothermic steam reforming reactions.

Direct air gasification is the utmost investigated of these, compassing a gas product with a comparatively low heating value because of the diluting result of nitrogen.

Gasification with pure O_2_ is an alternative to air and steam that combines the benefits of both gasifying agents. Although, due to the operating costs and high capital assets for air separation, this choice is more expensive and complex for medium-size utilizations in particular (Xiao et al., 2007). Recently, pyrolysis–reforming (in-line) of pyrolysis volatiles has been intended as a favorable waste plastics H_2_ production valorization route (Czernik and French, 2006; Wu and Williams, 2010a; Park et al., 2010; Namioka et al., 2011; Barbarias et al., 2016a; Barbarias et al., 2016b). Furthermore, this alternative makes use of highly active reforming catalysts, which enable the production of tar-free syngas, overcoming the key problem in standard gasification of plastics.

### 6.1 Steam gasification

Plastic steam gasification has received little attention in the literature. In contrast to air gasification studies, which have almost entirely been conducted in fluidized bed reactors, plastics waste steam gasification has been investigated in various reactor types (Table 1), such as fluidized beds (FBRs) (Martínez-Lera et al., 2013a), fixed (packed) bed (HeMXiao et al., 2009a; Wang et al., 2012; Friengfung et al., 2014; Lee et al., 2014), and conical spouted beds reactors (CSBRs) (Erkiaga et al., 2013a; Lopez et al., 2015b). Heat requirement and the content of tar in the product gas are the challenges that steam gasification faces. To overcome this limitation, Wilk and Hofbauer (2013) investigated steam gasification of various plastics in a dual fluidized bed reactor, with a 100-kW pilot plant. At 850°C, the gasification reactor runs an *in situ* primary catalyst of olivine with an S/P ratio of 2.

**TABLE 1:** Different gas compositions obtained by authors in steam plastics waste gasification.

Plastic type	Reactor	Reaction conditions	Bed material	Composition of gas (% vol)	Gas produced (m^3^/kg)	LHV (MJ/m^3^)	Tar content (g/m^3^)	References
Waste plastics	Plasma reactor	Gasifying agent: steam/O_2_ T: 1,200	—	CO: 34, H_2_: 62, CH_4_: –, CO_2_: –	3.5	10.1	—	Rutberg et al. (2013b)
PE	Spouted bed reactor (0.1 kg h^−1^)	T: 900, S/P: 1	Olivine	CO: 27, H_2_: 58, CH_4_: 7, CO_2_: 3	3.2	16.2	15	Erkiaga et al. (2013a)
PE	Spouted bed reactor	T: 900, S/P: 1	γ-Alumina	CO: 26, H_2_: 59, CH_4_: 8, CO_2_: 2	3.3	16.2	16.1	Erkiaga et al. (2013a)
PE	Two steps: Spouted bed plus packed bed reactor (0.1 kg h^−1^)	T: 900/600–700, S/P: 1	Olivine/NiCa-Al_2_O_4_	CO: 8–12, H_2_: 71–73, CH_4_: 3–0.3, CO_2_: 17–15	4.4–5.6	—	0	Lopez et al. (2015b)
PET	Semi-batch and fixed (packed) bed reactor	T: 1,000	—	CO: 6, H_2_: 61, CH_4_: 2, CO_2_: 12	—	7.8	—	Lee et al. (2014)
PS + PE	Fluidized bed (dual) (15 kg h^−1^)	T: 850, S/P: 1.8	Olivine	CO: 24, H_2_: 52, CH_4_: 12, CO_2_: 7	1.4	17	110	Martínez-Lera et al. (2013a)
PET + PE	Fluidized bed (dual) (15 kg h^−1^)	T: 850, S/P: 1.2	Olivine	CO: 20, H_2_: 27, CH_4_: 15, CO_2_: 29	1	16.4	160	Martínez-Lera et al. (2013a)
PE + PP	Fluidized bed (dual) (15 kg h^−1^)	T: 850, S/P: 2.0	Olivine	CO: 22, H_2_: 46, CH_4_: 16, CO_2_: 5	2.1	19.4	30	Martínez-Lera et al. (2013a)
PP	Fluidized bed (dual) (15 kg h^−1^)	T: 850, S/P: 2.0	Olivine	CO: 4, H_2_: 34, CH_4_: 40, CO_2_: 8	1	27.2	180	Martínez-Lera et al. (2013a)
PE	Fluidized bed (dual) (15 kg h^−1^)	T: 850, S/P: 2.0	Olivine	CO: 7, H_2_: 38, CH_4_: 30, CO_2_: 8	1.2	25.8	190	Martínez-Lera et al. (2013a)
HDPE	Fixed (packed) batch bed (0.1 g)	Gasifying agent: steam/O_2_ 1:1, T: 850	Ni-dolomite	CO: 43, H_2_: 35, CH_4_: 11, CO_2_: 10	2.4	—	17	Friengfung et al. (2014)
PS	Fixed (packed) batch bed (0.1 g)	Gasifying agent: steam/O_2_ 1:1, T: 850	Ni-dolomite	CO: 43, H_2_: 29, CH_4_: 1.7, CO_2_: 26	1.3	—	290	Friengfung et al. (2014)
PP	Fixed (packed) batch bed (0.1 g)	Gasifying agent: steam/O_2_ 1:1, T: 850	Ni-dolomite	CO: 45, H_2_: 38, CH_4_: 9, CO_2_: 8	1.9	—	140	Friengfung et al. (2014)
Plastic waste	Fixed (packed) batch bed (0.1 g)	T: 850 (15 °C/min)	—	CO: 19, H_2_: 44, CH_4_: 20, CO_2_: 13	—	20.4	—	Akkache et al. (2016)
Plastic waste and refuse paper	Fixed (packed) batch bed (0.1 g)	T: 900	—	CO: 22, H_2_: 38, CH_4_: 12, CO_2_: 17	0.9	17.9	—	Hwang et al. (2014)
PW waste	Fixed (packed) bed (0.3 kg h^−1^)	T: 700–900, S/P: 1.33	Ni/γ-Al_2_O_3_	CO: 20–27, H_2_: 17–37, CH_4_: 21–10, CO_2_: 35–21	1.22–2.04	12.4–11.3	106–13	HeMXiao et al. (2009a)

Erkiaga et al. (2013a) investigated the HDPE steam gasification in a spouted bed conical continuous bench scale reactor (0.1 kg/h) operating at temperatures ranging from 800 to 900°C. Operating at temperatures above 850°C and with an S/P of 1, the product stream H_2_ content was slightly higher than 60%, accounting for an 18 wt% production. Because of the decrease in hydrocarbon content, the gas heating value decreased from 19.3 to 15.4 MJ/m^3^ as the gasification temperature was raised. At the highest temperature investigated for an inert sand bed, a minimal tar content of 16.8 g/m^3^ was obtained, and this tar was interestingly composed primarily of single-ring aromatics. In the syngas, the tar content was slightly reduced and had little effect on the gas composition, by utilizing γ-alumina and olivine as the primary catalysts. The same authors used Ni reforming commercial catalyst with a fixed-bed (packed) reactor connected in-line with the spouted conical bed gasifier in a subsequent study (Lopez et al., 2015b). The operating temperature of the fixed bed is between 600 and 700°C with gasification experimental parameters being the same as those used in a previous study. The production of H_2_ increased up to 36.5 wt% by the addition of a catalytic reforming step and also enabled the full reforming of tar and hydrocarbons.

The gasification of PP and PE generates syngas with up to 40% H_2_ concentration, accordingly with 4–3 wt% of H_2_ production rates (gH_2_ 100 g/plastic). However, the most notable aspect of the composition gas product were the high concentrations of CH_4_ (40% and 30%, respectively) and C_2_H_4_ (11% and 15%) in the PP and PE gasification. The heating value of the produced gas up to 25 MJ/m^3^ due to high hydrocarbon content. However, the high concentration of light hydrocarbons and methane as previously investigated by other authors is a clear indication for the presence of tar (Pohorelyl et al., 2006; Mastellone and Arena, 2008; Pinto et al., 2009a; Mastellone et al., 2010a), and for both plastics, the values of tar content were higher than 120 g/m^3^, with naphthalene as the prevailing compound. In utilizing the same experimental parameters in biomass gasification, lower tar values have been reported by the same authors (Schneider et al., 2022).

Using an Ni-Al_2_O_3_ catalyst, HeMXiao et al. (2009a) investigated the PE gasification (0.3 kg/h) with 1.33 S/P ratio between 700 and 900°C in a fixed (packed) bed reactor. The production and concentration of H_2_ improved significantly to 3.7 and 6.6 wt%; conversion of plastic improved with temperature increase; and at 900 C, gases' yield reached 2.04 m^3^/kg. On steaming after 3 h time, no deactivation was evident by reforming the (Ni-based) catalyst. The gas product heating value ranged from 12.3 to 11.4 MJ/m^3^, at the lowest temperature, with the highest value being obtained.

Dou et al. (2016) recently conceived a laboratory-scale continuous reaction system consisting of a fluidized bed (FBR) gasifier followed by CO_2_/steam reforming adsorption in a moving bed reactor. The combination of steam reforming on CO_2_ retention on CaO and a Ni-Al_2_O_3_ catalyst resulted in the high production of H_2_; however, they discovered that below 700°C, adsorption of CO_2_ was only effective.

**FIGURE 7:**
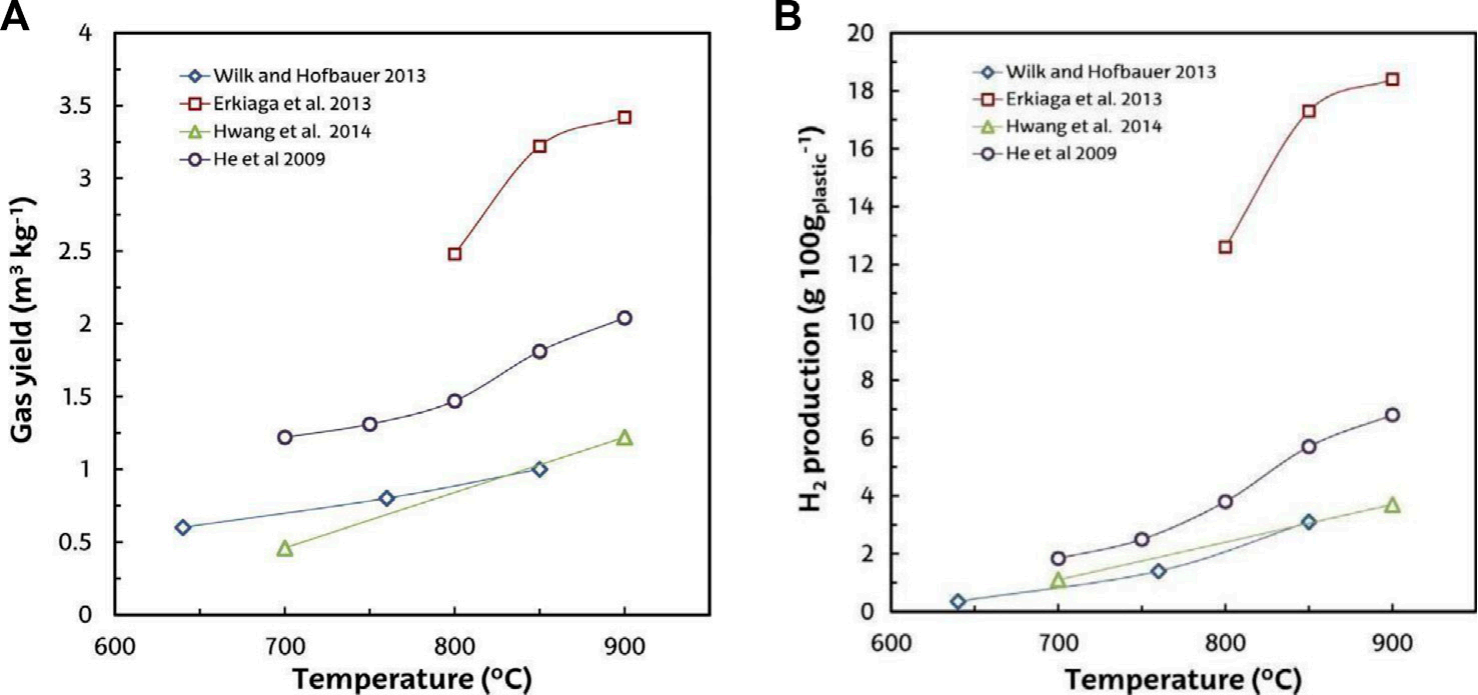
**(A)** Temperature effect on yield of gas in steam plastic waste gasification. **(B)** Temperature effect on H_2_ production.

In the literature, the values of H_2_ production with high concentrations of H_2_ vary between 3 and 18 wt% (g 100 g/plastic) of polyolefins steam gasification (HeMXiao et al., 2009a; Erkiaga et al., 2013a; Martínez-Lera et al., 2013a). Furthermore, the syngas obtained is suitable for the synthesis of various fuels (methanol, DME, and hydrocarbons) (Zhang, 2010). Temperature is the most critical and important parameter in the steam gasification of plastics. Its increase facilitates the cracking and reforming of endothermic reactions that include tar and light hydrocarbons, which facilitates the yield of both gas (Figure 7A) and H_2_ (Figure 7B). However, for synthesis applications in the gaseous stream, the tar content must be considerably decreased to achieve stringent tar content constraints (Devi et al., 2003). Steam gasification of plastic waste, as previously reported, results in high concentrations of tar in the gas product, even exceeding 100 g/m^3^ (HeMXiao et al., 2009a; Martínez-Lera et al., 2013a). In fact, it is widely acknowledged that air gasification results in less tar than that obtained through steam gasification (Gil et al., 1999; Devi et al., 2003), and as compared with the gasification of biomass and coal, the gasification of plastic waste yields more tar (Pinto et al., 2009b; Mastellone et al., 2010b; Martínez-Lera et al., 2013a).

By using fixed-bed batch reactor, Friengfung et al. (2014) studied the laboratory-scale gasification of steam/O_2_ (0.1 g of sample) of various plastics. By utilizing (Ni-impregnated) dolomite and dolomite at 850°C, the experiments were carried out without a catalyst. In all cases, the tar production was higher (more than 80 wt%) and the results obtained without a catalyst with various polyolefins, PP, LDPE, and HDPE were poor. In the HDPE case, promising results were achieved by utilizing Ni-impregnated dolomite catalyst for which a tar production of 10 wt% or below was achieved. The gasification efficiency is enhanced by utilizing a dolomite catalyst, but the tar production was on the higher side (more than 50 wt%). For full-scale development, steam gasification faces considerable challenges due to its high process heat requirement, however N_2_ absence improves the gas heating value over 15 MJ/m^3^ (Erkiaga et al., 2013a; Martínez-Lera et al., 2013a; Hwang et al., 2014). In fact, the well-designed dual fluidized beds scheme is also scarce by the low fixed carbon of waste plastics, which impedes the heat balance closure process (Wilk and Hofbauer, 2013; Schneider et al., 2022). Generally, steam gasification of waste plastics has received little attention and development and is not as advanced and promising when compared to air gasification.

### 6.2 Air gasification

The main challenge of gasification of plastic processes, regardless of the gasifying agent utilized, is the yield of the gas product tar, though when O_2_ or air is utilized in the place of steam, the tar yield is lower (Gil et al., 1999; Devi et al., 2003). Thus, the content of tar must be less than 10 mg N/m^3^ for the utilization of syngas for the production of energy in turbines and engines but much lower for synthesis applications (Devi et al., 2003). Deposition in the process equipments, especially in heat exchangers, and the characteristics of tar, mainly its dew point, play a vital role in the problems that it causes (Guan et al., 2016). The dew point is determined by the amount of tar present, and its composition, since single-ring aromatic hydrocarbons are non-condensable even at concentrations of 10 g N/m^3^. At the concentration of just 1 mg N/m^3^, polyaromatics with more than four rings condense, resulting in serious operational problems (Anis and Zainal, 2011).

Air gasification studies on plastic waste have primarily been conducted in fluidized bed reactors (FBRs), with substantial advancement in experimental units, especially the bench scale or pilot plants functioning in a continuous mode. Table 2 summarizes the important outcomes in air gasification of plastic waste. Air gasification has been broadly examined by the research group of Prof. Arena. They used plastic mixtures and different plastics in a pilot plant fluidized bubbling bed with a surmised capacity between 30 and 100 kg/h (Mastellone and Arena, 2008; Arena et al., 2009; Arena et al., 2010; Arena et al., 2011; Arena and Di Gregorio, 2014). Their early research focused on PE waste gasification with equivalence ratios (ERs) in between 0.21 and 0.33 at 850°C to investigate the role of olivine as the main catalyst for tar diminution (Mastellone and Arena, 2008; Arena et al., 2009). The gasification process efficiency improved significantly with the use of olivine, resulting in significant tar content reduction in the product gas. This result is linked not only to direct tar cracking but also to the removal of its promoters, i.e., light olefins. By improving the reforming reactions, the composition of the gas was also improved, resulting in a significant increase in H_2_ content. Thus, in experiments using inert silica powder, the content of tar in the product gas was about 100 g N/m^3^, while when calcined olivine was utilized *in situ* as the catalyst, the tar was almost completely removed. The efficiency of carbon conversion, or the fraction of carbon in the feed that is altered into products in the stream outlet, has been shown to increase the overall process output when olivine is used. At low ERs, this parameter increased by 60%–66%, while at high ERs, it increased by 70%–82%. In the gas product, the increase in equivalence ratio had a positive impact on the content of tar yield. However, the dilution effect due to the increased gas output for high ER values may also be a factor. The same authors have equated the gasification efficiency of various plastic waste mixtures retrieved from MSW and postconsumer packaging in a subsequent study (Arena et al., 2010). The *in situ* waste gasification of a mixture of polyolefin with olivine yields a gas fraction composition, process efficiency, and tar yield that are close to those which have result with pure PE, demonstrating the versatility of this valorization path. Poor results however have been obtained in the case of complex plastic mixture gasification with low process efficiencies and high tar yields. This is due to the reduction in the performance of the primary olivine catalyst.

**TABLE 2:** Gas compositions achieved by various researchers in air gasification of plastics waste.

Plastic type	Reactor	Reaction Conditions (°C)	Bed material	Composition of gas (% vol)	Gas produced (m^3^/ kg)	LHV (MJ/m^3^)	Tar Yield (g/m^3^)	References
Plastic waste	Fixed (packed) bed (0.06 kg/h)	T:700–900, ER: 0.4	-	CO: 0.2–4, H_2_: 0–2, CH_4_: 21–20, CO_2_: 5–7	-	7.8–8	18–12	Kaewpengkrow et al. (2012)
Mixture of waste plastic	Moving grate * fueled with pure O_2_ (80 kg/h)	T:700–900, ER: 0.15–0.6	-	CO: 22–33, H_2_: 41–29, CH_4_: 4.3–10, CO_2_: 8.2–22	1.2-1.5	9.0–11.8	-	Lee et al. (2013)
PE	Bubbling fluidized (aggregative) bed (100 kg/h)	T: 845–897, ER: 0.20–0.31	Sand	CO: 2.8–2.2, H_2_: 9.1–9.5, CH_4_: 10.4–7.1, CO_2_:9.1–10.4	3–4.3	7.9–6.3	160–81	Arena et al., (2010)
PE	Bubbling fluidized (aggregative) bed (100 kg/h)	T: 807–850, ER: 0.2–0.29	olivine	CO: 18.4–20.9, H_2_: 30.1–29.1, CH_4_: 3.4–1.5, CO_2_:1.6–1.2,	4.2-6.2	7.6–6.3	0	Arena et al., (2010)
Mixture of waste plastic	Bubbling fluidized (aggregative) bed (100 kg/h)	T: 869–914, ER: 0.22–0.31	Olivine	CO: 3.7–4.8, H_2_: 6.8–6.6, CH_4_: 7.3–6.3, CO_2_: 11.1–11.6	2.5-3.2	6.8–5.2	99-56	Arena et al., (2010)
Mixed waste (polyolefins)	Bubbling fluidized (aggregative) bed (100 kg/h)	T: 887, ER: 0.25	Olivine	CO: 4.5, H_2_: 5.9, CH_4_: 6.6, CO_2_: 10.3	3.3	6.6	59	Arena and Di Gregorio, 2014
Mixed cellulosic and plastic waste	Bubbling fluidized (aggregative) bed (100 kg/h)	T: 869, ER: 0.24	Olivine	CO: 6.6, H_2_: 6.0, CH_4_: 6.5, CO_2_: 12.7	2.73	7.4	34	Arena and Di Gregorio, 2014
Recycled plastic waste from packaging	Bubbling fluidized (aggregative) bed (5 kg/h)	T: 887, ER: 0.25	Silica sand	CO: 6.6, H_2_: 6.0, CH_4_: 6.5, CO_2_: 12.7	3.5	7.9	46	Zaccariello and Mastellone, 2015
PP	Fluidized bed (FBR) (1/kg h)	T: 850, ER: 0.32–0.36	Sand	CO: 5, H_2_: 5, CH_4_: 3, CO_2_: 12	4.5	2.9	17	Sancho et al. (2008)
PP	Fluidized bed (FBR) (1/kg h)	T: 850, ER: 0.32–0.36	70% sand−30% dolomite	CO: 7, H_2_: 6, CH_4_: 8, CO_2_: 16	5.3	7.4	1.5	Sancho et al. (2008)
PP	Fluidized bed (FBR) (1/kg h)	T: 850, ER: 0.32–0.36	70% sand−30% olivine	CO: 4, H_2_: 5, CH_4_: 7, CO_2_: 14	2.9	5.8	10	Sancho et al. (2008)
PP	Fluidized bed (FBR) (1/kg h)	T: 850, ER: 0.32–0.36	olivine	CO: 8, H_2_: 10, CH_4_: 7, CO_2_: 11	6	6	2	Sancho et al. (2008)
PP	Fluidized bed (FBR) (4/kg h)	T: 690–950, ER: 0.2–0.45	bottom ash	CO: 20–15, H_2_: 4–5, CH_4_: 6–4, CO_2_: 9–15	2-3.8	11.3–5.2	40-1.3	Xiao et al. (2007)
Mixture of plastic waste	Fluidized bed plus fixed bed (0.5 kg/h)	T: 800/830, ER: 0.2	olivine/active carbon	CO: 6.7, H_2_: 27.1, CH_4_: 6.4, CO_2_: 8.5	-	5.8	-	Cho et al. (2013b)
Mixture of plastic waste	Fluidized bed plus fixed bed (0.5 kg/h)	T: 800/800, ER: 0.2	silica sand/dolomite	CO: 6.6, H_2_: 14.2, CH_4_: 15.7, CO_2_: 4.0	-	13.4	-	Kim et al. (2011)
Mixture of plastic waste	Fluidized bed plus fixed bed (0.5 kg/h)	T: 800/800, ER: 0.2	silica sand/active carbon	CO: 6.7, H_2_: 15.2, CH_4_: 14.8, CO_2_: 4.5	-	13.2	-	Kim et al. (2011)
PE	Bubbling fluidized (aggregative) bed (1 kg/h)	T: 750, ER: 0.3	silica sand	CO: 6.1, H_2_: 2.7, CH_4_: 7.0, CO_2_: 8.8	3.6	3.9	128	Martínez-Lera et al. (2013b)
Polyolefins waste	Bubbling fluidized (aggregative) bed (1 kg/h)	T: 750, ER: 0.25–0.35	silica sand	CO: 8.5–10, H_2_: 3, CH_4_: 8.5-10, CO_2_: 7.8–6.5	3.2–4.4	4.9–5.7	150-55	Martínez-Lera et al. (2013b)
PE waste	Bubbling fluidized (aggregative) bed (1 kg/h)	T: 750, ER: 0.3	silica sand	CO: 8.7, H_2_: 3, CH_4_: 8.7, CO_2_: 7.4	3.7	4.9	102	Martínez-Lera et al. (2013b)

In a bench scale two-step unit, Kim et al. (2011) investigated air gasification with a continuous feed rate of 0.50 kg/h of plastic waste mixture composed of polyolefins and other waste plastics (PET, PVC, and PS). Both phases were conducted at about 800°C in fluidized bed reactors, with the first containing sand, followed by the second, i.e., tar cracking catalysts. Dolomite and activated carbon were among the catalysts investigated, with activated carbon proving to be a better option for tar removal. Apart from reducing the tar content, the utilization of activated carbon as a primary catalyst significantly improved the content of H_2_ in gas products. Based on experimental parameters, the tar yields ranged from 3 to 7 wt% with the impact of catalytic bed mass being particularly noticeable. The same authors suggested a similar approach in a subsequent study conducted under similar conditions, but they substituted sand with olivine in the first bed and dolomite as the primary cracking catalyst (Cho et al., 2013a; Cho et al., 2013b). The fraction of gas composition improved significantly with the utilization of dolomite. Furthermore, combining both these catalysts in the first bed with active carbon in the second bed provided a tar yield of less than 2 wt%. In bubbling fluidized bed gasifier (4 kg/h bench scale), Xiao et al. (2007) investigated the impact of various operating variables like equivalence ratio, gas velocity, and residence time on PP air gasification. The presence of Fe, Al, Mg, and Ca caused tar cracking activity in the bottom ash from a boiler. The most important variable analyzed was ER, which induced a substantial increase in the temperature of the gasifier from 705 to 917 °C when ER was increased from 0.23 to 0.47. Furthermore, in the ER range investigated, the gas product tar content decreased from 40.3 to 0.25 g N/m^3^. A higher yield of gas and the high temperature were both responsible for this reduction. For high ER values, the authors found that the equivalence ratio should be thoroughly calibrated to prevent a decrease in the heating value of the gas product.

At 850°C, Sancho et al. (2008) studied PP air gasification in a continuous fluidized bed reactor (bench scale) with an equivalence ratio of about 0.35 at 1 kg/h. This study evaluated the catalytic efficiency of dolomite and olivine as the primary catalysts and compared the findings to those procured with inert sand. They found that the use of dolomite is restricted by its low physical ability, which drives it to be ejected from the gasifier. Moreover, olivine has material characteristics that make it ideal for use in fluidized beds, with a catalytic activity that is just marginally lower than dolomite. As a result of the use of olivine, the content of tar in the product gas was decreased from 17 g N/m^3^ achieved with sand to 2 g N/m^3^. Furthermore, olivine facilitates reforming hydrocarbon reactions, which increases the amount of hydrogen in the syngas. The same authors went on to investigate the use of olivine in PP air gasification, demonstrating olivine permanence over long gasification runs (Toledo et al., 2011). Furthermore, the values of the equivalence ratio were dropped from 0.37 to 0.24 to increase the heating value of the gas product while maintaining the tar content at a low. This was accomplished by raising the gasifier freeboard region temperature up to 915°C by using an external heat source.

**FIGURE 8:**
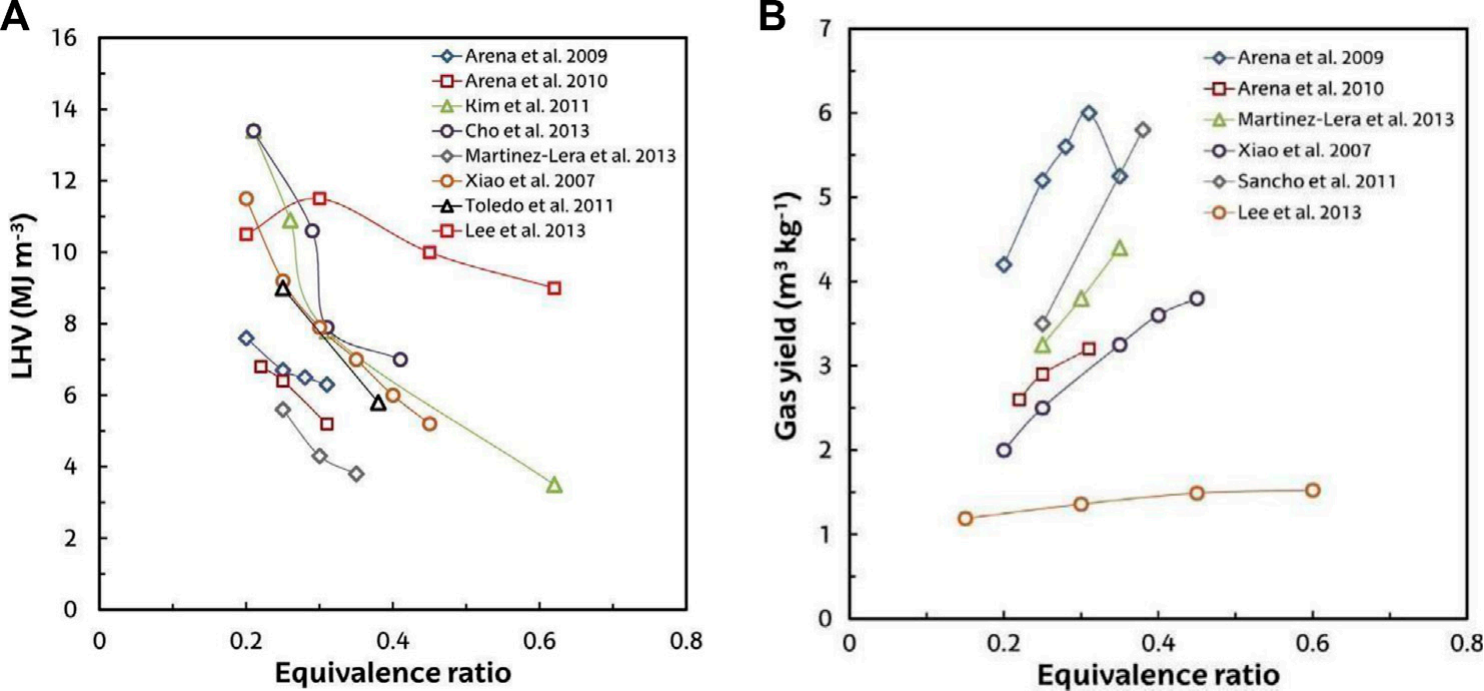
**(A)** ER effect on gas LHV product in air plastic waste gasification. **(B)** ER effect on the yield of gas by Lee et al. (2013).

In a moving grate pilot plant gasifier, Lee et al. (2013) studied the gasification of waste plastics with an output of 80 kg/h. The gasifying agent used was pure oxygen. Under these parameters, the ideal equivalence ratio was between 0.30 and 0.45, and the gas yield was from 1.35 to 1.48 m^3^/kg with the heating value above 10 MJ/m^3^. Plastic waste air gasification is an intriguing option for producing a gas stream adequate for a variety of energy applications, the most viable one being electricity generation in engines and turbines (Heikkinen et al., 2004). As shown in Figure 8A (ER 0.2 and 0.45), the heating value is 3–12 MJ/m^3^ of produced gas. This heating value is primarily influenced by two factors: 1) equivalence ratio and 2) waste plastics composition. In the gasification of plastic waste, the heating value (average) is approximately 6–8 MJ/m^3^ (Table 2).

The air gasification of pure PP, PE, and PE waste has been studied by Martínez-Lera et al. (2013c) in a bubbling fluidized bed bench-scale gasifier with a capacity of 1 kg/h. The bed was composed of inert silica sand with an equivalence ratio of 0.25–0.35, and the experiments were carried out at 750°C. Pure PP and PE gasification produced similar gas compositions and yields. However, waste PE gasification produced better results than pure polyolefins gasification. As a result, the gas yield achieved with PE waste was 92.7%, while that of pure PE was 90.6%, with the tar content difference being more substantial. The tar content obtained from pure PE and waste plastics was 127 g N/m^3^ and 103 g N/m^3^. Despite the fact that the ER was only changed to a small degree (0.25–0.35), it had a significant impact on the process efficiency, especially tar yield. As a result, it was lower from around 150 g N/m^3^ to below 60 g N/m^3^ in the case of PE waste. A semiempirical model was developed by Martínez-Lera and Pallarés Ranz (2017) for polyolefin in FBR gasification, with the model predictions confirmed by previously described findings and others from the literature.

The equivalence ratio is undoubtedly the most significant parameter in terms of impact on air gasification operating conditions since it specifies the composition and yield of the gas (Xiao et al., 2007; Martínez-Lera et al., 2013c). Increased ER contributes to higher gas production, but it also reduces the gas heating value (Figure 8B). In the gas product, the presence of N_2_ increases with an increase in ER value, and the combustion of CH_4_, CO, and H_2_ and the resulting increase in CO_2_. An increase in the ER usually reduces the gas product tar content, which not only increases the gasifier temperature but also the volumetric gas yield.

The gasifier’s design is also essential for improving tar removal quality. To favor the cracking of tar in FBRs, an increase in temperature and residence time in the freeboard area is typically sought (Toledo et al., 2011; Martínez-Lera et al., 2013b). In fluidized bed gasifiers, the feed location also affects the tar yield (Wilk et al., 2013; Brachi et al., 2014). Secondary air injections in the gasifier’s free board are another popular technique for improving tar cracking and increasing the temperature in this region (Narváez et al., 1996; Pan et al., 1999). In a plastic waste air gasification, the amount of tar in the gas produced by different researchers varies greatly and depends on various factors, i.e., catalyst utilization, design of the reactor, the composition of plastics, and experimental parameters, in particular residence time, temperature, and equivalence ratio (Table 2). In general, the contents of tar are higher than biomass gasification (Pinto et al., 2009b; Mastellone et al., 2010b; Pinto et al., 2016), whose average value in the FBR reactors is 10 g/m^3^ (Anis and Zainal, 2011).

Since the content of tar has a significant impact on the direct use of the gas generated, various strategies for eliminating or reducing it have been suggested. As a result, using a primary catalyst *in situ* reduces the tar content of the gas component substantially (Sancho et al., 2008; Arena et al., 2009; Toledo et al., 2011). While in tar cracking, dolomite is more effective than olivine (Rapagna et al., 2000; Corella et al., 2004; Sancho et al., 2008; de Andres et al., 2011). In FBR reactors, olivine is the more commonly utilized catalyst because of its refined mechanical characteristics (Sancho et al., 2008; Arena et al., 2010; Toledo et al., 2011; Arena and Di Gregorio, 2014). The olivine catalytic function is generally linked to the content of iron (II) oxide (Kumar and Singh, 2011), with interest stemming from not only the enhanced removal of tar promoters but also the ability of the catalyst in the direct cracking of tar, preventing further formation of tar in the gasification system (Arena et al., 2010; Schneider et al., 2022). Different catalysts, like active carbon (Kim et al., 2011; Cho et al., 2013a; Cho et al., 2013b), zeolite (Cho et al., 2014), dolomite (Kim et al., 2011; Cho et al., 2013b), and active carbon filled with Ni (Cho et al., 2015), have been proposed for catalytic cracking of tar in secondary beds. Furthermore, for the removal of tar from the gas product, electrostatic precipitators and filters have been recommended (Kim et al., 2011; Cho et al., 2013a).

### 6.3 Co-gasification

The degree to which the product distribution is dependent on the composition of the feed is a notable differentiation between gasification and pyrolysis processes. As a result, the yield and composition of the products derived from pyrolysis of various solid wastes are extremely different. Moreover, the variations in gasification of various feed materials are limited to the composition of gas and small byproduct yields such as char and tar. The analysis of waste plastics co-gasification has been aided by the flexibility of the gasification process, and the higher advancement level of the gasification of biomass and coal.

Pinto et al. (2003) used a fluidized bed gasifier (5.5 kg/h) to investigate coal air/steam co-gasification with lower concentrations of PE and biomass (20% each). Plastic co-feeding increased the hydrocarbon content in the product gas; however, this result could be prevented by working at higher ERs or temperatures. A similar pattern was observed in the formation of tar. In order to achieve an appropriate performance for each mixture of feedstock, they found that the gasifier operating parameters had to be thoroughly calibrated. The same authors were able to fully eliminate tar by using two secondary fixed-bed (packed) tar cracking reactors, the first of which used dolomite and the second of which used Ni-Al_2_O_3_ (Pinto et al., 2009b). Surprisingly, holding unwanted halogen and sulfur mixtures in the dolomite bed bettered the durability and performance of the Ni-based catalyst. Pinto et al. recently investigated rice husk co-gasification (80%)/PE (20%) in a fluidized bed gasifier utilizing various gasifying agents, such as air, pure oxygen, steam, and mixtures of these agents (Kaewpengkrow et al., 2012; Pinto et al., 2016). The findings show that working with steam and pure oxygen produces the best gas, but that the usage of pure oxygen is restricted due to high oxygen production cost, thereby considering enriched air as a viable alternative.

Pinto et al. (2002) investigated the PE/biomass mixture steam gasification (continuous) in an FBR. The PE maximum content studied (60%) resulted in effective conversion, as demonstrated by a particular gas yield and a heating value, i.e., 1.96 kg/m^3^ and 18.3 MJ/m^3^. Furthermore, an increase in PE feed resulted in an increase in methane concentrations and H_2_ (to 52%) on the one side, but a decrease in CO_2_ and CO concentrations on the other.

Despite the fact that plastic waste has mostly been co-gasified with biomass (Pinto et al., 2002; Wilk and Hofbauer, 2013; Alvarez et al., 2014; Narobe et al., 2014; Lopez et al., 2015a; Zaccariello and Mastellone, 2015; Arena and Di Gregorio, 2016; Singh et al., 2022), it has also been co-processed with ternary mixtures (Ahmed and Gupta, 2011; Jung et al., 2013; Lopez et al., 2016) and coal (Mastellone et al., 2010b; Kriz and Bicakova, 2011; Straka and Bicáková, 2014b). Steam, air, or their mixtures were used as the gasifying agent in these experiments. Table 3 summarizes the key findings in the co-gasification of plastic waste.

**TABLE 3:** Gas compositions obtained by authors in the plastics waste co-gasification.

Plastic type	Reactor	Bed material	Gasifying agent	Reaction Conditions (°C, -)	Composition of gas (% vol)	Gas yield (m^3^/kg)	LHV (MJ/m^3^)	Tar yield (g/m^3^)	Reference
PE(0.3)/wood pellets (0.7)	Fluidized bed (dual) (15 kg/h)	olivine	steam	S/F: 1.6, T: 850	CO: 23, H_2_: 41, CH_4_: 14, CO_2_: 16	1.9	16	47	Wilk and Hofbauer, (2013)
MSW plastic(0.5)/wood pellets (0.5)	Fluidized bed (dual) (15 kg/h)	olivine	steam	S/F: 0.94, T: 850	CO: 24, H_2_: 35, CH_4_: 6, CO_2_: 19	1.1	16	39	Wilk and Hofbauer, (2013)
PE(0.33)/lignite (0.66)	Fluidized bed (dual) (15 kg/h)	olivine	steam	S/F: 0.90, T: 850	CO: 24, H_2_: 45, CH_4_: 8, CO_2_: 10	-	13	9	Kern et al. (2013)
Wood (0.2)/recycled plastic (0.8)	Bubbling Fluidized (aggregative) bed (5 kg/h)	SiO_2_	air	T: 872, ER: 0.25	CO: 7, H_2_: 10, CH_4_: 8, CO_2_: 11	3.4	7	34	Zaccariello and Mastellone, (2015)
wood (0.2)/Recycled plastic (0.3)/ coal (0.5)	Bubbling Fluidized (aggregative) bed (5 kg/h)	SiO_2_	air	T: 868, ER: 0.25	CO: 13, H_2_: 14, CH_4_: 2, CO_2_: 14	2.7	6	41	Zaccariello and Mastellone, (2015)
Wood(0.5)/HDPE(0.5)/PE (0.5)	Spouted (conical) bed reactor (0.1 kg/h)	olivine	steam	S//F: 1.00, T: 900,	CO: 27, H_2_: 57, CH_4_: 6, CO_2_: 7	2.64	-	9.7	Lopez et al. (2015a)
PE(0.5)/coconut shell (0.5)	Fluidized bed/fixed (packed) bed (2/kg)	Commercial Ni catalyst/dolomite	steam	S/F: 2, T: 800/600	CO: 9, H_2_: 82, CH_4_: 7, CO_2_: 2	2.7	12.4	0	Alipour Moghadam Esfahani et al. (2017)
PE (0.5)/rice straw (0.5)	Fixed (packed) bed	-	steam	T: 900	CO: 30, H_2_: 46, CH_4_ :12 CO_2_: 12,	1.1	13.9	-	Baloch et al. (2016)
Wood and biomass paper fiber(0.45)/ waste polyolefins (0.55)	Updraft (60kg/h)	-	air	ER: 0.19-0.24, T: 800–930	CO: 15–14, H_2_: 10–15, CH_4_: 6-5, CO_2_: 8	2.6-3.4	9.5-79	22-11.2	Ponzio et al. (2006)
Biomass (0.5)/PP (0.5)	Dual Fixed bed (0.04g)	Fe-CeO_2_	steam	T: 850/700	CO: 5, H_2_: 40, CH_4_: 6, CO_2_: 16	2.55	35.5	-	Parparita et al. (2015)
PET (0.5)/wood (0.5)	Fluidized (heterogeneous) bed reactor	olivine	air	ER: 0.19-0.31, T: 725-875	CO: 13-9, H_2_: 4.3-5.4, CH_4_: 3-2.7, CO_2_: 17	-	4.5-3.5	145-63	Robinson et al. 2016
PE(0.2)/ rice husk (0.8)	Fluidized (heterogeneous) bed reactor (0.3 kg/h)	-	oxygen	ER:0.20, T:850	CO: 12, H_2_: 38, CH_4_: 12, CO_2_: 37	1	13	12	Pinto et al. (2016)
PE(0.2)/ rice husk (0.8)	Fluidized (heterogeneous) bed reactor (0.3 kg/h)	-	air	ER:0.20, T:850	CO: 24, H_2_: 19, CH_4_: 13, CO_2_: 33	1.3	8	12	Pinto et al. (2016)
PE(0.2)/ rice husk (0.8)	Fluidized (heterogeneous) bed reactor (0.3 kg/h)	-	steam	S/F: 1, T:850	CO: 15, H_2_: 41, CH_4_: 11, CO_2_: 24	0.35	13	15	Pinto et al. (2016)
PE (0.1)/ pine wood (0.9)	Fluidized (heterogeneous) bed reactor (0.75 kg/h)	-	steam	S/F: 0.8, T: 740–880	CO: 34–31, H_2_: 25–44, CH_4_: 15–10, CO_2_: 14–9,	0.63–1.28	21–15	-	Pinto et al. (2002)
PE (0.1)/ coal (0.9)	Fluidized (heterogeneous) bed reactor (6 kg/h)	-	steam/air	S/F: 0.85, ER: 0.2, T: 850	CO: 17, H_2_: 40, CH_4_: 17, CO_2_: 16	1.3	-	19	Pinto et al. (2009b)
PE (0.2)/pine wood (0.2)/coal (0.6)	Fluidized (heterogeneous) bed reactor (5.5 kg/h)	-	steam/air	Air/ F: 1.14, S/F: 1, T: 740–880	CO: 18–17, H_2_: 25–40, CH_4_: 18–15, CO_2_: 24–20,	0.6–1.35	24–18	-	Pinto et al. (2003)
PE (0.2)/ pine wood(0.8)	Fluidized (heterogeneous) bed reactor (5 kg/h)	quartz sand	air	ER: 0.23, T: 780	CO: 16, H_2_: 17, CH_4_: 12, CO_2_: 15	-	7.3	60	Ruoppolo et al. (2012)
PE (0.2)/ pine wood(0.8)	Fluidized (heterogeneous) bed reactor (5 kg/h)	Ni-γAl_2_O_3_	air	ER: 0.23, T: 780	CO: 14, H_2_: 30, CH_4_: 3 CO_2_: 10	-	6.5	27	Ruoppolo et al. (2012)
Polyolefins waste (0.4)/ coal (0.6)	Fluidized (heterogeneous) bed reactor (4 kg/h)	sand-dolomite	air	ER: 0.36, T: 850	CO: 22, H_2_: 40, CH_4_: 5.5, CO_2_: 16	2.9	8.3	1.3	Aznar et al. (2006)
biomass (0.2)/ polyolefins waste (0.2)/coal (0.6)	Fluidized (heterogeneous) bed reactor (4 kg/h)	sand-dolomite	air	ER: 0.36, T: 850	CO: 12, H_2_: 11, CH_4_: 2, CO_2_: 14	3	5.5	1	Aznar et al. (2006)
PET (0.25)/ olive husk (0.75)	Fluidized (heterogeneous) bed reactor (5 kg/h)	γ-Al_2_O_3_	steam/air	S/F: 0.76, ER: 0.1, T: 752	CO: 13, H_2_: 33, CH_4:_ 9, CO_2_: 19	1.3	10.2	90	Brachi et al. (2014)
PET (0.25)/ olive husk (0.75)	Fluidized (heterogeneous) bed reactor (5 kg/h)	Ni-γAl_2_O_3_	steam/air	S/F: 0.62, ER: 0.1, T: 845	CO: 22, H_2_: 40, CH_4_: 5.5 CO_2_: 16	1.4	9	29	Brachi et al. (2014)

At 900°C in a laboratory fixed-bed batch reactor, Ahmed and Gupta (2011) studied PE steam co-gasification and wood chips. In the co-processing of biomass and plastics, they also discussed the synergistic impact on gas yields, hydrocarbons, and hydrogen, as well as on thermal performance. Furthermore, in the feed, the optimized content of plastic was found to be within 65 and 80%. Lopez et al. (2015a) confirmed the previously recorded synergistic effects in a spouted bed conical gasifier (0.1 kg/h) using biomass and PE co-gasification. This effect is particularly noticeable at a 1/1 blending ratio.

The gas product tar content of a 1/1 mixture of biomass and PE gasification was decreased to 9.5 N/m^3^ with an S/F ratio of 1, by utilizing a primary olivine catalyst operating at 900 °C. Furthermore, while the gas yield (2.67 kg/m^3^) was close to the theoretical value predicted in accordance with the results achieved for biomass and PE particular feeds, a synergistic impact on the char yield reduction and H_2_ content in the syngas was observed.

By utilizing olivine as the bed material in the dual fluidized bed gasifier (15 kg/h), Wilk and Hofbauer (2013) investigated biomass pellets steam co-gasification with various waste plastics types and their mixtures (such as PE). Thus, a 16 MJ/m^3^ LHV value of 1.6 m^3^/kg gas yield was reported for 1/1 ratio of blended HDPE/biomass, which is significantly less than that obtained with pure plastic. Moreover, when plastics and biomass were co-gasified, a synergistic effect on the formation of tar was observed, with the tar contents being less than that predicted based on their particular gasification. Furthermore, tar composition was also affected by an increase in the content of plastic in the feed thus lowering furan and phenol while enhancing naphthalene content. Similarly, by utilizing different blending ratios, non-linear patterns were perceived, and the composition of the gas product cannot be directly evaluated from the outcomes achieved with particular feedstocks. The impact of lignite co-feeding in the PE steam gasification was investigated by the same authors (Kern et al., 2013). Furthermore, lignite co-feeding was found to have a synergistic impact on cold gas efficacy, and lignite co-feeding also enabled a reduction in the content of tar when contrasting with those results from pure plastic.

In a fluidized bed pilot scale gasifier, Ruoppolo et al. (2012) explored pellets gasification containing 20% PE and 80% wood, and correlated the results to those from pure biomass. Ni-Al_2_O_3_ and inert quartzite catalyst were utilized as bed materials. Mixtures of air and air/steam were utilized as gasifying agents, and they discovered that by improving the reforming reactions, air/steam mixtures resulted in a higher hydrogen concentration and a lower content of tar. The high concentration of H_2_ obtained during PE pellets gasification was the most promising result (30% vol.). Despite their utilization of comparatively low Ni-Al_2_O_3_ catalyst and the content of plastic in the pellets, the tar content as compared with biomass (below 30 g N/m^3^) was significantly higher (around 46 g N/m^3^). Therefore, the above synergies in steam gasification were apparently less pronounced when air was used as the gasifying agent. The same authors investigated gasification of pellets composed of olive PET (25%) and husk (75%) with mixtures of steam/air, but with low ERs to increase syngas efficiency (Brachi et al., 2014). When a nickel-based catalyst (Ni-Al_2_O_3_) was compared with an Al_2_O_3_ catalyst, the former produced better gas composition and tar content. Furthermore, when the effects of feeding from a bed middle point were compared to those from the top bed feeding, a substantial increase in gasifier efficiency was observed.

A two-step gasification framework was developed by Park et al. (2016) that included oxidative pyrolysis at 526°C and a plasma thermal reactor operating at 626°C. Different mixing ratios and equivalence ratios were used to investigate the biomass and HDPE co-gasification. With an ER of 0.46 and 70% biomass in the feed, the best results were achieved.

In a fluidized bed pre-pilot gasifier, Mastellone et al. (2010b), Mastellone et al. (2012), and Zaccariello and Mastellone (2015) investigated the air gasification of ternary mixtures composed of biomass, coal along with plastic mixtures, and coal. Because of the higher light hydrocarbon content, the key result of plastics co-feeding was an improvement in heating value and gas yield. When plastics were used in the feed, they found a rise in tar formation and a decrease in H_2_ concentration, i.e., for various ERs (0.21–0.31), the co-gasification of coal/plastics tar contents ranged from 26 to 48 gm^−3^. Surprisingly, biomass had the opposite effect than that predicted, which is tar formation reduction. As a result, the authors assessed that by promoting synergistic effects in the feed by using appropriate component proportions, the process’ viability can be increased.

Moghadam et al. (2014) and Alipour Moghadam Esfahani et al. (2017) proposed a two-stage method for HDPE steam gasification and a palm kernel shell/coconut shell mixture between 660 and 880°C in FBR using *in situ* Ni catalyst (powder), followed by cracking of tar in an FBR dolomite reactor at 600 °C. This method produces syngas with high H_2_ content and allows for effective tar removal. Hence, at the maximum gasification (880 °C) temperature, a hydrogen yield of 29.4 wt% was recorded, with an 87% concentration (by vol).

Furthermore, plastics in the feed had a positive effect on the content of tar and gas heating value; but on tar formation, this effect was found to be the opposite of that stated by other authors (Ruoppolo et al., 2012; Wilk and Hofbauer, 2013; Zaccariello and Mastellone, 2015). Thus, for binary and ternary mixtures, very low contents of tar (1.35 gm^−3^) were achieved by operating at 850°C, utilizing dolomite as the primary catalyst and with an ER of 0.36, with heating values in the range of 5–8 MJ/m^3^ due to the high equivalence ratio used.

Aznar et al. (2006) studied air co-gasification of binary and ternary mixtures in a fluidized bed reactor. The mixtures were made up of plastic waste, i.e., PP and PE, biomass, and coal. In binary mixtures, the content of plastics was comparatively high (40%), while in ternary mixtures, the content was low (10–20%). The concentration of hydrocarbon in the gas production increased due to the presence of plastics in the feed while lowering H_2_, CO_2,_ and CO.

**FIGURE 9:**
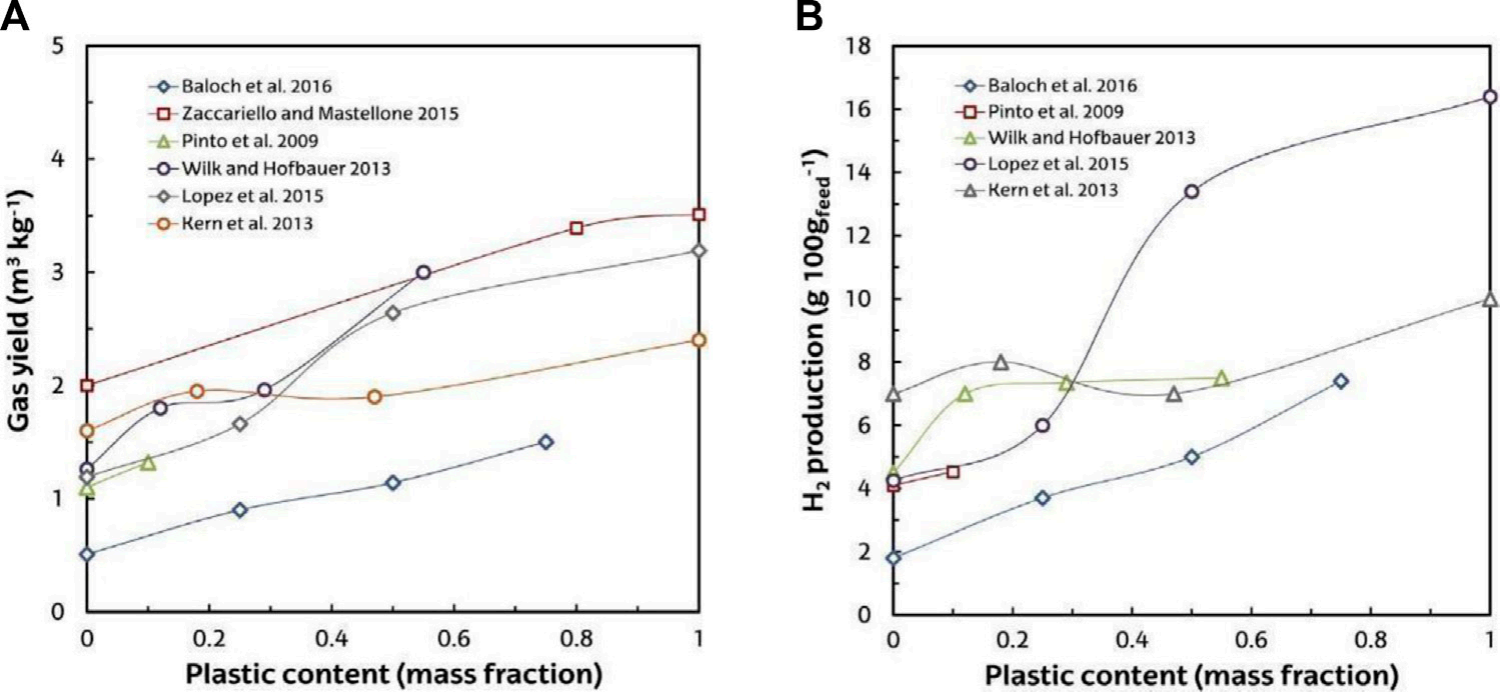
**(A)** The effect of the feed’s plastic content on the yields of gas in plastics co-gasification with coal and biomass. **(B)** Plastic content effect in the feed on the production of H_2_ in plastics co-gasification with coal and biomass.

**FIGURE 10:**
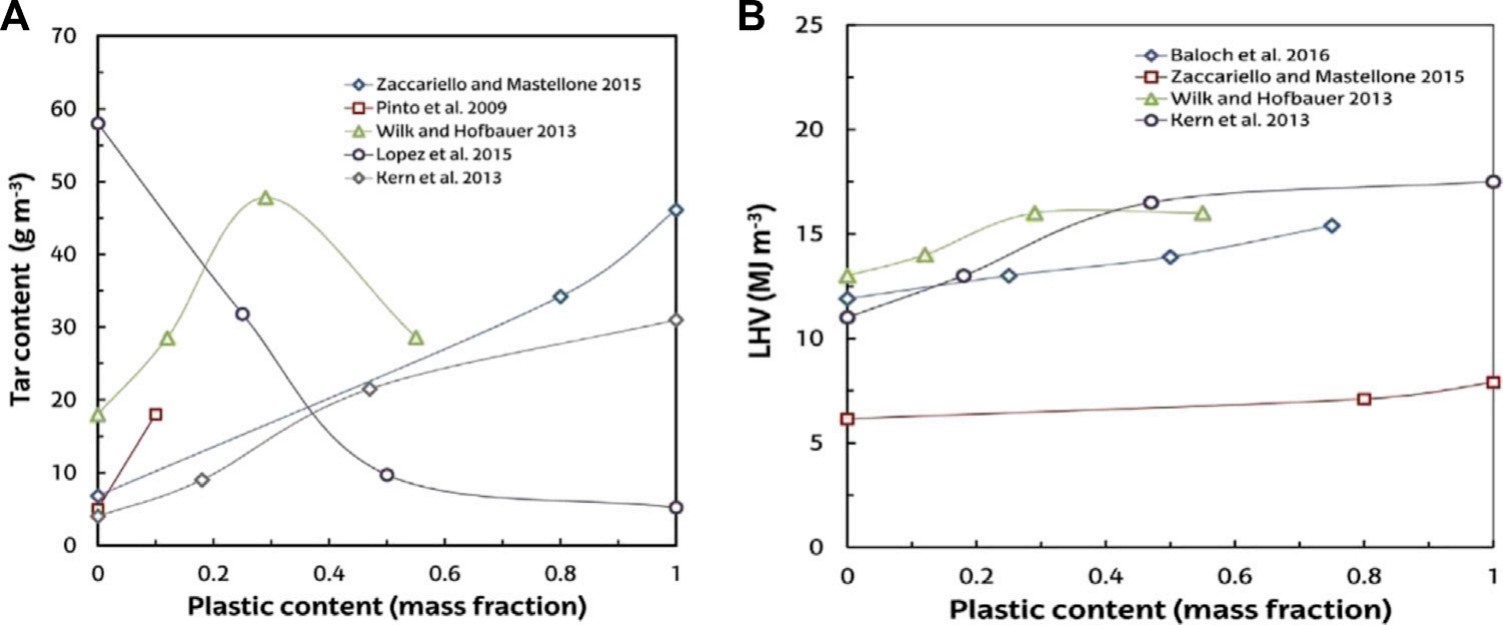
**(A)** Plastic content effect in the feed on the content of tar in the produced gas in plastics co-gasification with coal and biomass. **(B)** Plastic content effect in the feed on the produced gas heating value in plastics co-gasification with coal and biomass.

According to these results, plastic waste co-gasification with various feedstocks produces fascinating synergies, highlighting the strategy’s utility (Wilk and Hofbauer, 2013; Lopez et al., 2015a; Singh et al., 2022). The reciprocations between product polymer degradation and biomass chars are usually due to these synergies (Antelava et al., 2021), with a positive correlation in their thermal joint degradation being well established (Zhang et al., 2016b; Lopez et al., 2017). As shown in Figures 9A,B, increasing the content of plastics in its co-gasification with coal and biomass increases both H_2_ concentration and gas yield. These outputs are explicated by the higher content of carbon and H_2_ in waste plastics when compared to coal and biomass, as well as the lower or non-existent char yield. An increase in the formation of tar is the key plastics co-feeding disadvantage as shown in Figure 10A. The higher gas heating value generated when compared to that in the gasification of biomass, as shown in Figure 10B, also facilitates the plastic co-feeding benefit (Pinto et al., 2016). Another benefit of co-gasification of plastic and biomass is that this reduces plastics gasification operational issues, such as formation of fine char particulates and reactor feeding (Pinto et al., 2002).

## 7 Pyrolysis

Pyrolysis is organic matter thermal decomposition without oxidizing agents like CO_2_, oxygen, or steam. The temperature for pyrolysis processes is generally inbetween 300 and 850°C, depending on various process parameters. Usually, pyrolysis processes are endothermic, which means that energy is required to proceed with the process. The energy content and composition of pyrolysis products are dependent largely on the input of waste and can differ significantly (Hu et al., 2021; Tezer et al., 2022):

• Solid: a char-like substance that contains residual solid products, such as sand, glass, and metals. The heating values and char content (by weight) are around 10–35 MJ/kg and 20–50%, respectively, which may have substantial content of ash (10–50%).
• Liquid: a complex mixture of hydrocarbons, such as organic acids, phenols, PAHs, and alcohols, made up of water, tar, and oil. The heating values and liquid amount (by weight) are around 5–15 MJ/kg and 30–50%.
• Gas: a mixture of CO, CH_4_, CO_2_, H_2_, and other volatile waste constituents. The heating value and gas yield may be around 3–12 MJ/Nm^3^ and 20–50%, respectively.

Moisture is released and waste is dried during the pyrolysis process, which involves heating the waste to about 100–120°C. Following this process, a series of complex reactions take place, resulting in the release of volatile compounds and the breakdown of more complex carbon-containing compounds into simpler ones. Gaseous outputs are formed by breaking nitrogen, hydrogen, and oxygen bonds at temperatures ranging from about 200°C to 800 C (see Table 4). The primary reactions are those that result in the production of gas and tar/oil, while the secondary reactions are those that result in the conversion of gas and tar/oil. During gasification, these secondary reactions can also occur. Secondary reactions convert further tar to gases and char, along with the enhancement in the concentrations of CH_4_ and CO_2_ in the gas product.

**TABLE 4:** Temperature-dependent pyrolysis reactions (Bilitewski et al., 1997).

Chemical reaction	Temperature range
Dehydration, thermal drying	100–120
Desulfurization, deoxidation, CO_2_ and H_2_O molecular splitting, H_2_S splitting	250
Aliphatic hydrocarbon bonds breakage, methane and other aliphatic hydrocarbons splitting	340
Carbonization	380
C-O and C-N bonds breakage	400
Bituminous (asphalt) compounds disintegration into low temperature tars and oils	400–600
Bituminous (asphalt) compounds cracking into thermal resistant elements, aromatic organic compounds formation	600
Thermal aromatization of ethene to hexanaphthene to C_6_H_6_ formation and other volatile aromatic hydrocarbons	>600

Pyrolysis product heating values and mass yields differ greatly from one process to another and also depend highly on the composition of the waste input. With well-sorted solid recovered fuel (SRF), automotive shredder residue (ASR), or biomass waste as a process input, the above values can only be considered suggestive and typically representing an upper limit. Mixed plastics generally produce high amounts of inorganic residues and char, whereas high quality plastic waste and rubber promote higher oils and gases ratios.

The amount of water in the waste input has an impact on both the process conditions and outputs, especially on the liquid and gas outputs. Heat is mostly supplied indirectly *via* the reactor walls, but waste compaction and friction can also lead to waste heating. Pyrolysis takes place in an inert atmosphere, but in practice, it occurs in the pyrolysis gaseous atmosphere that go through various secondary conversion reactions.

## 8 Pyrolysis of waste plastics

Plastics come in a variety of compositions which are usually stated based on their proximate analysis. The proximate analysis includes the determination of volatile matter, moisture content, fixed carbon, and waste sample ash content. If samples of solid waste are to be utilized as a fuel, all of these characteristics are very significant (Kreith, 1998). The main factors that affect the yield of liquid oil in the pyrolysis process are ash content and volatile matter. A high content of ash decreases the liquid oil yield, while high volatile matter enhances the production of liquid oil (Abnisa and Wan Daud, 2014). The proximate study of various plastics is summarized in Table 5, which shows that all plastics have high volatile matter and low ash content. These properties show that plastics have a high capacity for pyrolysis to produce significant amounts of liquid oil.

**TABLE 5:** Proximate analysis of plastics (Abnisa and Wan Daud, 2014).

Plastics types	Marks on plastics	Volatile (wt%)	Fixed carbon (wt%)	Ash (wt%)	Moisture (wt%)	References
Polyethylene terephthalate (PET)	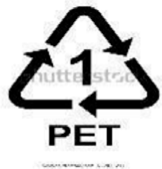	91.75	7.77	0.02	0.46	Zannikos et al. (2013)
		86.83	13.17	0	0.61	Heikkinen et al. (2004)
High-density polyethylene (HDPE)	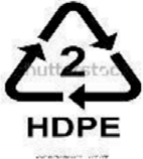	99.81	0.01	0.18	0	Ahmad et al. (2013)
		98.57	0.03	1.40	0	Heikkinen et al. (2004)
Polyvinyl chloride (PVC)	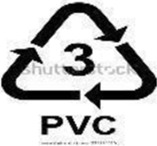	93.70	6.30	0	0.80	Hong et al. (1999)
		94.82	5.19	0	0.74	Heikkinen et al. (2004)
Low-density polyethylene (LDPE)	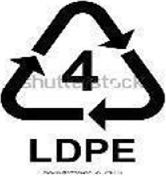	99.70	0	0	0.30	Park et al. (2012)
		99.60	-	0.40	-	Aboulkas et al. (2010)
Polypropylene (PP)	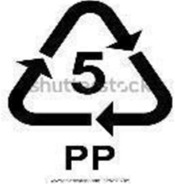	95.08	1.22	3.55	0.15	Jung et al. (2010)
		97.85	0.16	1.99	0.18	Heikkinen et al. (2004)
Polystyrene (PS)	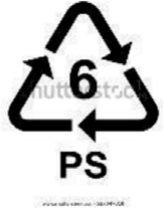	99.63	0.12	0	0.25	Abnisa et al. (2014)
		99.50	0.20	0	0.30	Park et al. (2012)
Polyethylene (PE) Acrylonitrile butadiene styrene (ABS) Polyamide (PA) or Nylons Polybutylene terephthalate (PBT)	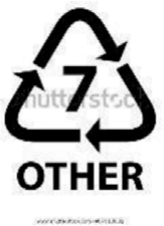	98.87	0.04	0.99	0.10	Jung et al. (2010)
		97.88	1.12	1.01	0	Othman et al. (2008)
		99.78	0.69	0	0	Othman et al. (2008)
		97.12	2.88	0	0.16	Heikkinen et al. (2004)

### 8.1 High-density polyethylene

Polyethylene is the most popular plastic in the world. It is the most basic of all commercial thermoplastics in terms of structure. Its molecules are made up of long-chain carbon atoms joined by two atoms of hydrogen. The straight chain (no branching) is called high-density polyethylene (HDPE) or linear PE, short for high-density polyethylene. Although linear PE is far more durable than branched PE, branched PE is easier to manufacture and less expensive. Its different uses account for 17.6% of the plastic waste group, which is the third most common plastic form of MSW (Michael, 2010). HDPE is therefore suitable for applications like weaving, Raschel knitting, reinforcement applications, and braiding. Many studies on the pyrolysis of HDPE at various operating conditions have been performed to determine the yield of the product.

Using a batch reactor, Marcilla et al. (2009a) explored the pyrolysis of HDPE at 550°C. The gaseous product produced was 16.4 wt% and the yield of liquid oil was 84.8 wt%. The findings showed that at higher temperatures, more liquid oil yield could be produced, but there was also a drawback that should be observed; since the process had reached the utmost thermal decomposition stage, too high temperatures would increase the gaseous product while decreasing the yield of liquid oil. Mastral et al. (2001) studied the pyrolysis of HDPE at 650 °C in an FBR. During experimentation, they noted that the production of the gaseous product was 31.7 wt% and liquid oil yield was 68.3 wt%. They found that when the temperature exceeds 550°C, the liquid further cracks into the gaseous products.

Kumar and Singh (2011) investigated the thermal pyrolysis of HDPE at 400–550°C utilizing a semi-batch reactor. At 550°C, gaseous product (24.73 wt%) and the maximum liquid yield (79.06 wt%) were obtained, while at temperatures of 500–550°C, wax began to dominate the fraction of the product. The pyrolysis produced a dark brownish oil with no clear residue and a boiling point ranging from 83 to 351°C. This indicated that the oil contained a mixture of components of various oils, like diesel, kerosene, and gasoline, which coordinated the characteristics of conventional fuel (see Table 6). In addition, the pyrolytic oil of HDPE had a very low sulfur content (0.018%), making it environmentally friendly.

**TABLE 6:** Properties comparability of conventional fuel and pyrolytic HDPE oil.

Oil type	Properties of conventional fuel (Boundy et al., 2011)		Characteristics of HDPE pyrolysis oil (Kumar and Singh, 2011)	
	Boiling point (°C)	Cv (MJ/kg)	Boiling point (°C)	Cv (MJ/kg)
Gasoline	40–200	42.9	82–352	43.4–46.5
Diesel	150–390			42.8–45.8
Kerosene	150–300			43.0–46.2

In a micro steel reactor, Ahmad et al. (2014) explored the pyrolysis of HDPE by utilizing nitrogen as a fluidizing medium at 5–10°C/min heating rate at 300–400°C. They discovered that the maximum total conversion occurred at 350°C, with liquid yield as the primary product (80.83 wt%). At 300°C, the solid residue was fairly significant (33.07 wt%), but it decreased to 0.53 wt% at the maximum temperature of 400 °C.

### 8.2 Low-density polyethylene

Low-density polyethylene (LDPE) is a semi-rigid, translucent plastic polymer. It has a large proportion of long and short side-chain branching than HDPE. Tubular and stirred autoclave processes are the two most used methods for producing LDPE. Because it has greater rates of ethylene conversion, the tubular method is becoming more popular than the autoclave method. Squeeze bottles, containers, carrier bags, wash bottles, laboratory molded apparatus, and high-frequency insulation are among the most common uses for LDPE. Plastic bags are the most common use for LDPE, therefore day by day, LDPE waste has been accrued and is now the second most used plastic after PP in MSW (Michael, 2010). Apart from that, LDPE also has the potential for energy recovery, i.e., converting it into liquid and gaseous products.

Uddin et al. (1996) investigated the pyrolysis of LDPE at 430 °C in a batch reactor. The yield of liquid product was about 75.7 wt%. By utilizing a similar reactor type as Uddin et al. (1996), Aguado et al. (2007) obtained a yield of 74.6 wt% at 450 °C which is closer to the yield obtained by Uddin et al. However, even at lower temperatures in the reactor, when pressure was applied during the operation, the yield of liquid oil could be increased. Onwudili et al. (2009) demonstrated this at 425 °C in LDPE pyrolysis using a pressurized batch reactor (0.7–4.2 MPa). They obtained 0.4 wt% char, 10 wt% gaseous products, and 89.6% liquid oil from the experiment. This suggests that pressure can have an effect on the pyrolysis product’s composition.

With a 10 °C/min heating rate, Bagri and Williams (2001) at 500 °C in a fixed-bed (packed) reactor studied the pyrolysis of LDPE by utilizing nitrogen as the fluidizing gas. During the experimentation, it was discovered that a 95% liquid yield was achieved with a low gas and char yield. Marcilla et al. (2009a) at 550 °C also investigate the LDPE pyrolysis in a batch reactor with a 5 °C/min heating rate. During experimentation, a high yield of liquid oil was obtained (93.2 wt%), while the gas yield was notably low.

### 8.3 Polyvinyl chloride

Polyvinyl chloride (PVC) is a thermoplastic resin that is widely utilized in the manufacturing of a wide range of products. PVC is a cost-effective and versatile polymer that is used in a variety of industries, such as the packaging, construction, automotive, and medical industries. PVC is different from other thermoplastics in terms that it is made up of a combination of carbon (43%) and chlorine (57%) (British Plastics Federation, 2015). Due to the content of chlorine in PVC, recycling it is more complex and challenging than recycling other polymers such as PET. To recycle PVC plastics, dechlorination is required.

In batch reactors under vacuum, Miranda et al. (1998) studied PVC pyrolysis at a 10°C/min heating rate, with applied pressure of 2 kPa, and at a temperature between 220 and 520°C. The accumulation of tar increased dramatically as the temperature increased and reached 19.5%, which was even higher than the liquid oil yield (12.78%). From the experiment, the primary product yield was hydrogen chloride (HCl) (58.32 wt%). When heated mildly, HCl is toxic and corrosive, resulting in equipment damage. This was one of the key reasons for the pyrolysis pilot plant in Germany (Ebenhausen), being shut down (Miranda et al., 1998). Therefore, PVC is not an ideal material for the pyrolysis process. There are two major reasons for this: firstly, PVC waste accumulation in MSW is very less (less than 3%) (Michael, 2010), and secondly, the presence of HCl in the liquid product is very harmful to the process equipment due to its corrosive properties. PVC dechlorination is required to overcome the problem and to make the pyrolysis process effective. This is possible through various techniques like catalytic pyrolysis, adding adsorbents to PVC, and stepwise pyrolysis (López et al., 2011). As a result, when an extra dechlorination phase is necessary, the PVC pyrolysis requires an additional cost, which has been one of the industry’s drawbacks.

### 8.4 Polyethylene terephthalate

Polyethylene terephthalate (PET) polymer is utilized in several applications, such as sheets, packaging, and industrial parts. PET has outstanding mechanical strength, transparency, and gas barrier characteristics. Printing pads, electrical insulations, photographic films, and X-ray and magnetic tapes and films are some of the other uses of PET (Çepeliog˘ ullar Ö Pütün, 2013). PET is the most extensively used and highly recycled plastic in the world. As reported by the PET Resin Association (PETRA), the PET recycling rate in the EU is about 52%, whereas in the United States the rate is 31%.

The recycling rate in the United States dropped below 29% in 2016. Over 1.8 billion pounds of PET had been recycled in 2015 and was utilized to produce a range of products. PET containers are estimated to account for 1% of MSW in the United States, according to the EPA. As a result, other options for the recovery of PET, like the pyrolysis process, have been investigated, and the yield of products has been studied by a number of researchers. Cepeliogullar et al. (2013) studied the pyrolysis of PET in a fixed-bed reactor by using nitrogen as the sweeping gas at a 10 °C/min heating rate and at a temperature of 500°C. The authors found that the yield of liquid oil (23.2 wt%) was significantly lower than the gaseous product (76.90 wt%). There was no solid residue left after the process. As shown in Table 5, the volatile content of PET is 86.83%, which is relatively low when compared to that of other plastics, which is the main reason for the low liquid oil yield. Unfavorably, GC-MS (gas chromatography mass spectroscopy) studies have revealed that benzoic acid is the primary product in the oil composition, which is about 49.93%. The acidic characteristic of the pyrolysis oil is unfavorable because of its corrosiveness, which degrades the fuel efficiency (Cepeliogullar et al., 2013). Moreover, the benzoic acid content in pyrolysis oil is generally sublime and can clog the piping of heat exchangers, necessitating close monitoring if used on an industrial scale (Shioya et al., 2005; Wan Ho, 2015).

### 8.5 Polypropylene

Polypropylene (PP) is a crystalline, rigid, and tough polymer made from the monomer of propylene (or propene). It is a hydrocarbon resin with a linear structure. PP is a polymer that belongs to the family of polyolefin and is one of the top three most extensively utilized plastics in the world. PP is a material that can also be used as a fiber and plastic in the furniture market, the automobile industry, consumer goods, and industrial applications. Polypropylene accounts for around 24.3% of the total amount of plastics contained in MSW (Michael, 2010). The pyrolysis of polypropylene has been investigated by many researchers, which are given below.

Ahmad et al. (2014) investigated PP pyrolysis in a micro steel reactor with temperatures between 250 and 400°C. They found that at 300°C, the yield of liquid oil obtained was 69.82 wt%, which was the highest at this temperature with 98.66% of total conversion. They also noted that an increase in temperature (400°C) increased the solid residue (1.33–5.70 wt%) and decreased the conversion of the product (94.30%). This means that at higher temperatures, coke formation increases. Sakata et al. (1999) explored the pyrolysis of polypropylene at 380°C. They obtained an 80.10 wt% yield of liquid oil, along with a 6.6 wt% gaseous yield and 13.30 wt% solid residue. Fakhrhoseini and Dastanian (2013) also explored the pyrolysis PP at 500°C. They obtained a higher yield of liquid product (82.12 wt%), but an increase in temperature above 500°C decreased the production of liquid oil. Demirbas (2004) proved this by investigating PP pyrolysis in a batch reactor at a very high temperature of 740°C. The yield of liquid produced was 48.8 wt%, with 49.6 wt% of gaseous product and 1.6 wt% of solid residue.

### 8.6 Polystyrene

Polystyrene (PS) is a versatile material that can be utilized in a wide range of customer goods. Its common applications are in products that demand limpidity, such as in laboratory ware and food packaging. PP is used to produce electronics, appliances, toys, automobile parts, and gardening pots when mixed with different additives, colorants, or polymers. Recycling of PS can be achieved in thermal, chemical, and mechanical ways. For mechanical recycling, high-impact polystyrene (HIP) is a propitious material because, despite several processing cycles, its properties remain the same. The liquid and gaseous products production depend highly on the reaction conditions. For the production of both gaseous and liquid products, high-selectivity catalysts are used.

In an autoclave pressurized batch reactor, Onwudili et al. (2009) investigated the pyrolysis of PS for a duration of 1 hour at 300–500°C. The experimental pressure was 0.32 MPa–1.6 MPa, and the rate of heating was 10°C/min. They noted that at 452°C, the gas yield production was only 2.6%, while the production of liquid oil was very high and around 97.0 wt%. Liu et al. (1999) investigated PS pyrolysis at 450–700°C in a fluidized bed reactor. At 600°C, the highest amount of liquid oil (98.7 wt%) was obtained. But at 450°C, the production of liquid oil was also considerably high which was 97.6 wt%. Demirbas (2004) also studied the pyrolysis of PS in batch reactors at 581 °C. From the experiments, the highest yield of liquid product was 89.5 wt% which is less when compared to those obtained by Onwudili et al. (2009) and Liu et al. (1999). Therefore, PS is not a favorable material for the pyrolysis process at a high temperature because of its effect on the end products.

### 8.7 Mixed plastics

The pyrolysis process has a benefit over recycling in that it does not require a thorough sorting process. Many plastics are incompatible with one another in their cycling processes and cannot be recycled together. For instance, a small PVC contamination quantity in the stream of PET recycling can degrade the whole resin of PET, turning it brittle and yellow, necessitating reprocessing (Hopewell et al., 2009). This demonstrates that the recycling process is so vulnerable to pollutants that all plastics must be sorted by transparency, color, and resin type. The pyrolysis process, on the other hand, appears to be more viable because liquid oil can still be obtained from any sort of plastic present in the feedstock. Donaj et al. (2012) explored the pyrolysis of mixed plastics in a bubbling fluidized bed reactor at temperatures between 650°C and 730°C. The plastics mixture comprised of 24 wt% PP, 30 wt% HDPE, and 75 wt% LDPE. They noted that at 650°C, the yield of liquid oil was 48 wt%. This oil fraction, on the one hand, was composed of 52% heavy fractions that included carbon black, wax, and heavy oil. The yield of liquid oil at 730°C (44 wt%), on the other hand, contained a liquid light fraction of up to 70%. Therefore, higher temperatures facilitate gaseous or light hydrocarbon liquids. Therefore, the distribution of the product changes dramatically when the temperature is increased to a high extent.

Kaminsky et al. (1996) studied mixed plastic pyrolysis, approximately composed of 25% PS and 75% polyolefins (PP, PE). The product yield contained a small amount of chlorine which demonstrated the presence of PVC content in the mixture (1 wt%). The yield of liquid oil obtained was 48.4 wt% at 730°C in an FBR. Demirbas (2004) also investigated mixed plastic pyrolysis that involved PS and polyolefins (PE, PP). The solid and gaseous yields were about 2.2 and 35 wt%, respectively. The yield of liquid oil was around 46.6 wt%, which was very similar to the yield obtained by Kaminsky et al. (1996). The composition of liquid oil also contained small amounts of chlorine (4 ppm) which was due to the presence of PVC in the feedstock. However, the presence of chlorine did not affect the quality of the liquid oil because its content was below 10 ppm. Moreover, the majority of the chlorine content was found in the solid residue. Therefore, in order to get a quality liquid oil yield, the feedstock’s chlorine content could not exceed 1 wt%. From the above results, it may be observed that when compared to the pyrolysis of single plastics, mixed plastics pyrolysis produces lower than 50 wt% liquid oil. Nonetheless, the produced oil had a composition similar to that of pyrolysis of the single plastic, making it suitable for petrochemical refineries for further processing.

## 9 Chemical reactors for pyrolysis of plastic waste

In the pyrolysis process, the reactor type used has a profound influence on the catalysts and plastics mixing, heat transfer, reaction efficiency, and residence time in order to achieve the desired final end product. At a lab scale, most of the experimentation is done in a fixed bed, fluidized bed, continuous flow, CSBR, and batch reactors.

### 9.1 Fixed-bed (packed) reactor and fluidized bed reactor

**FIGURE 11:**
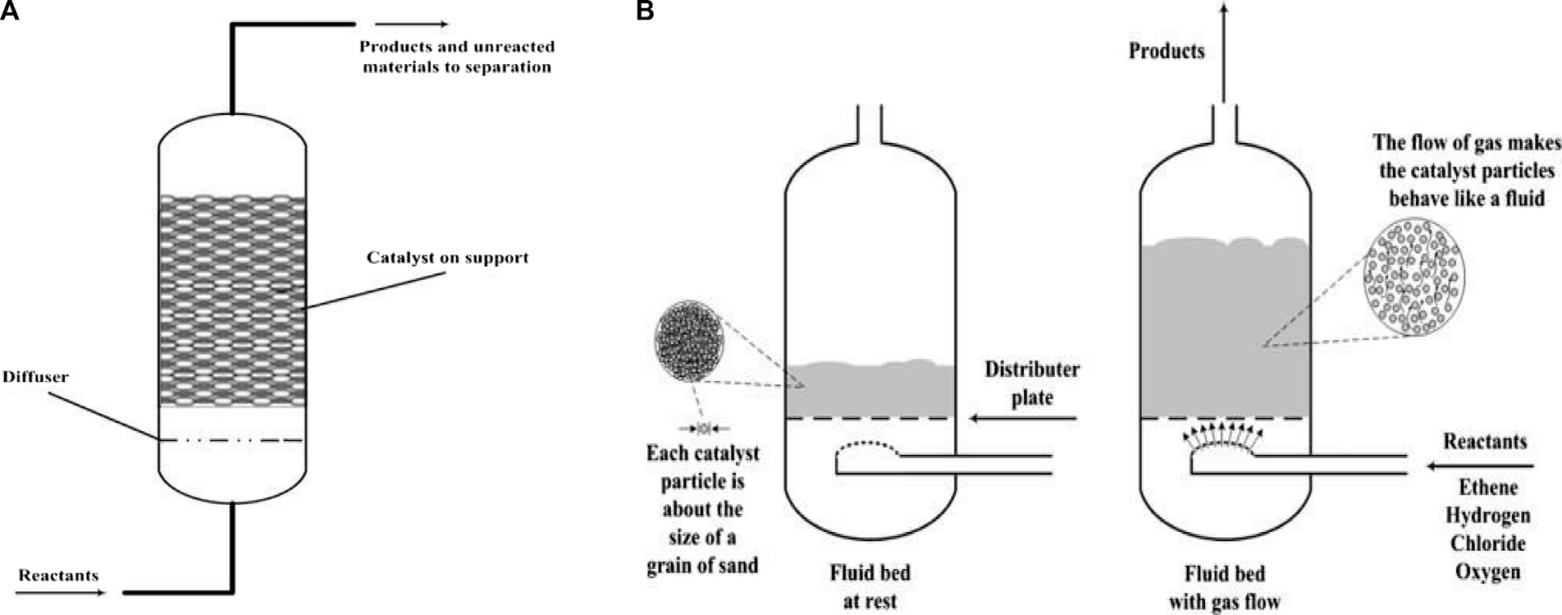
Representations of **(A)** fixed-bed (packed) reactor and **(B)** fluidized bed reactor (FBR) (The University of York, 2013).

The catalyst is normally packed and palletized in a static bed in a fixed-bed reactor, as shown in Figure 11A. The key advantage of these is their design simplicity, but on the other hand, there are some limitations, like the irregular shape and size of the plastic particles used as feedstock, which during the feeding process cause difficulties. Another disadvantage is that the reaction’s access to the catalyst’s usable surface area is limited. For the pyrolysis of plastic waste, many researchers have utilized the fixed-bed reactor (Bagri and Williams, 2001; Ballice, 2001; Choi et al., 2010; Renzini et al., 2011; Cepeliogullar et al., 2013; Saad et al., 2015a). Because it is easy to feed the primary pyrolysis product into the fixed-bed reactor, which is usually composed of gaseous and liquid phases, these reactors in some cases are only used as secondary pyrolysis reactors (Fogler, 2010). Onu et al. (1998) and Vasile et al. (2000) studied different plastic pyrolysis using a two-step procedure. The two-step process for plastic pyrolysis does not get much attention because it is not cost-efficient, and the product composition procured is equivalent to that obtained using the single-step process.

In plastics catalytic cracking, several studies have favored fluidized bed reactors to fixed-bed reactors (Sharratt et al., 1997; Garfoth et al., 1998; Williams and Williams, 1998; Liu et al., 1999; Mastral et al., 2001; Lin et al., 2004; Lin and Yen, 2005; Yan et al., 2005; Mastral et al., 2006b; Marcilla et al., 2007). Jung et al. (2010) studied the PE and PP pyrolysis processes in an FBR at temperatures between 290 and 850°C. The yield of liquid product was dramatically high because the reactor provides constant temperature with high heat and mass transfer, reliable mean time distribution, and uniform products spectrum. Luo et al. (2000) also studied the pyrolysis processes of PP and HDPE in an FBR at 500°C by utilizing a silica–alumina catalyst. The yield of liquid oil by HDPE was 85 wt%, while PP produced had a high liquid composition, which was 87 wt%.

In an FBR, unlike in a fixed-bed (packed) reactor, the catalyst sits on a distributor plate, as shown in Figure 11B, through which the fluidizing gas moves and the particulates are held in a fluid state. Since the catalyst is mixed thoroughly with the solvent, there is greater accessibility to the catalyst, resulting in a wider surface area for the reactions to take place (Kaminsky and Kim, 1999). With effective and viable heat transfer, this decreases process volatility. Furthermore, when compared to batch reactors, the FBR reactor is more flexible as it does not require regular feedstock charging, which makes the process steady. Therefore, because of the lower operating cost, the FBR will be the better reactor to use in the pilot plant on a traditional design scale.

Therefore, the FBR is more feasible to perform plastic catalytic degradation as it provides uniform catalyst mixing with the fluid, resulting in a high surface area for the reaction to take place. Furthermore, it does not require regular feedstock charging which makes the process steady. As a result, in terms of economics, the FBR will be the utmost appropriate reactor for large- and extensive-scale applications.

### 9.2 Batch and semi-batch reactors

Batch reactors are the most basic reactors used in chemical reactions. They are closed systems that work in an unsteady state, which means that no reactants or products inflow or outflow are possible during the reaction. In batch reactors, high residence time means higher conversion rate, which is one of their main advantages. The downsides of batch reactors are high labor cost and the difficulty in maintaining extensive production (Fogler, 2010). A semi-batch reactor, on the other hand, allows product removal and reactant addition at the same time. Concerning reaction selectivity, the semi-batch reactor has the advantage of being able to incorporate reactants over time. High labor cost and small-scale production are the main downsides of a semi-batch reactor.

Because of the easy configuration and ability to monitor the operating conditions readily, many researchers utilize batch and semi-batch reactors in the pyrolysis of plastic waste in laboratory-scale experiments (Cardona and Corma, 2000; Uemura et al., 2001; Kim and Kim, 2004; Miskolczi et al., 2004; García et al., 2005; Lee and Shin, 2007; Jan et al., 2010; Shah et al., 2010; Adrados et al., 2012; AdnanShah and Jan, 2014). The ideal temperature for catalytic and thermal pyrolysis in these reactors is in the range of 300–800°C. To increase the yield of hydrocarbons, many researchers have added catalysts to plastics. The main drawback of catalytic pyrolysis is the formation of coke on the catalyst surface which reduces the efficiency of the catalyst due to the blockage of its active sites, thus causing high residues during the reaction.

**FIGURE 12:**
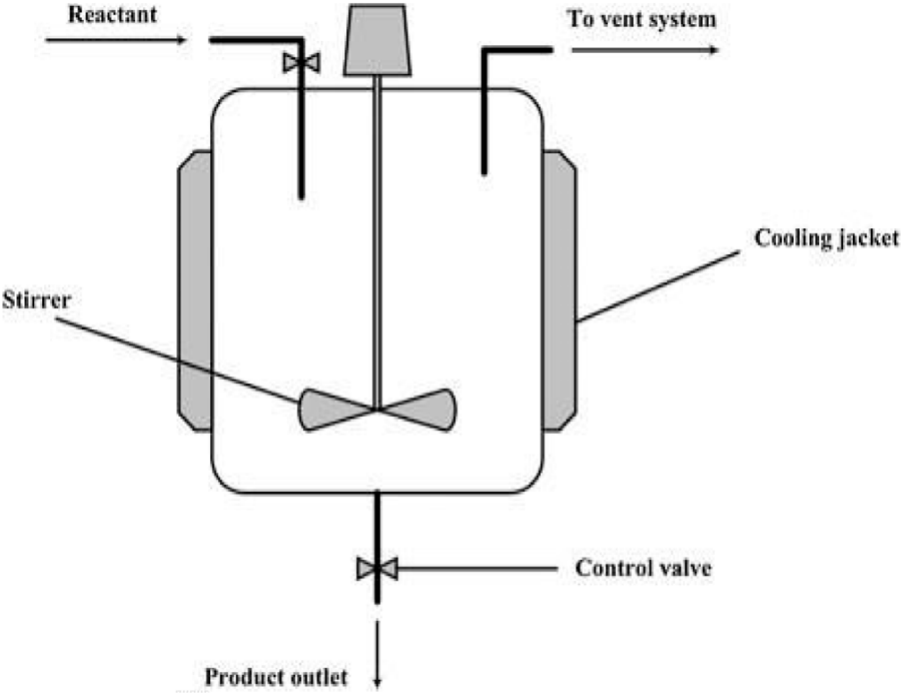
Batch reactor (sequential) with stirrer (The University of York, 2013).

Abbas-Abadi et al. (2014) studied the pyrolysis of PP in semi-batch reactors and found a very high liquid yield of 92.3 wt%. The experiment was conducted at 450°C using an FCC catalyst. As shown in Figure 12, some batch reactors and semi-batch reactors were also fitted with stirrers that ran at various speed depending on the necessary setting. Seo et al. (2003) explored the pyrolysis of HDPE at 450 °C by utilizing the stirrer batch reactor. The speed of the stirrer was 200 RPM. They found a high yield of liquid oil of 84.0 wt% in thermal pyrolysis than did Sakata et al. (1999). Furthermore, by using a silica–alumina catalyst, the liquid oil obtained by Sakata et al. (1999) was 74.3 wt%, while Seo et al. (2003) obtained a high liquid oil yield which was 78 wt%. As a result, it has become clear that in the batch reactor, the stirrer improved the mixing of plastics and catalysts within the reactor, thus increasing the yield of liquid oil. Kyong et al. (2002), Lee (20080, and Abbas-Abadi et al. (2013) conducted additional research on semi-batch reactors with stirrers. Sakata et al. (1999) studied the HDPE and PP processes with and without catalysts at 430°C and 380 °C, respectively, in batch reactors. For certain catalysts, the liquid oil yield through catalytic pyrolysis was even less than that obtained through thermal pyrolysis. The yield of liquid in thermal pyrolysis from HDPE was 69.4 wt%, and 80.2 wt% from PP. In catalytic pyrolysis, the yield of liquid for both plastics was decreased to 49.9–67.7 wt% (HDPE) and 47–78 wt% (PP). This might be due to the formation of coke on the catalyst surface which degraded the catalyst efficiency. The catalysts used in the experimentation were HZSM-5 and silica–alumina (SA-1). However, for both plastics, the liquid yield increased very slightly about 1.0–7.0 wt% than the thermal pyrolysis, by utilizing mesoporous silica and silica–alumina (SA-2) catalysts. Thus, the reactivity of various catalysts to various plastic types might be different. Based on the above results, it was found that the batch reactors and semi-batch reactors are favorable and feasible to be utilized in waste plastics pyrolysis process because it is easy to monitor the parameters of these reactors which promote the high yield of liquid. These reactors were however not appropriate for catalytic plastic pyrolysis due to the formation of coke on the outer surface of the catalyst which would affect the overall product composition. These reactors are only suitable for laboratory experiments because, on a large scale, it is difficult to maintain per unit of production.

### 9.3 Spouted bed reactors

The spouted bed reactor (CSBR) offers fine amalgamation and can accommodate a broad particle size distribution, different particle densities, and larger particles (Fogler, 2010). The CSBRs have been used by several researchers in the catalytic pyrolysis of plastics (Aguado et al., 2002; Elordi et al., 2009; Olazar et al., 2009; Arabiourrutia et al., 2012b; Elordi et al., 2012; Artetxe et al., 2013b). The CSBR, according to Olazar et al. (2009), have lower bed segregation and attrition when compared to the bubbling fluidized bed. The CSBR offers inconsiderable defluidization issues while processing sticky materials and also provides excellent heat transfer between the phases. However, the main downsides of this reactor are product collection, entrainment and feeding of the catalyst, and high operating cost (Lopez et al., 2009).

The CSBRs are particularly well suited for preventing problems of agglomeration in the polyolefins pyrolysis, even when the process is performed under maximum stickiness conditions. Aguado et al. (2005) investigated the LDPE, HDPE, and PP pyrolysis processes using the 1:30 g of plastic/sand ratio. The experiments were performed at 400, 500, 550, and 600°C. The authors found that, for a certain sand amount, the amount of plastic fed into the reactor increases almost linearly as the gas velocity rises, which results in increasing particle velocity. Moreover, the particles’ rapid velocity causes collisions that have enough energy to prevent agglomeration.

**FIGURE 13:**
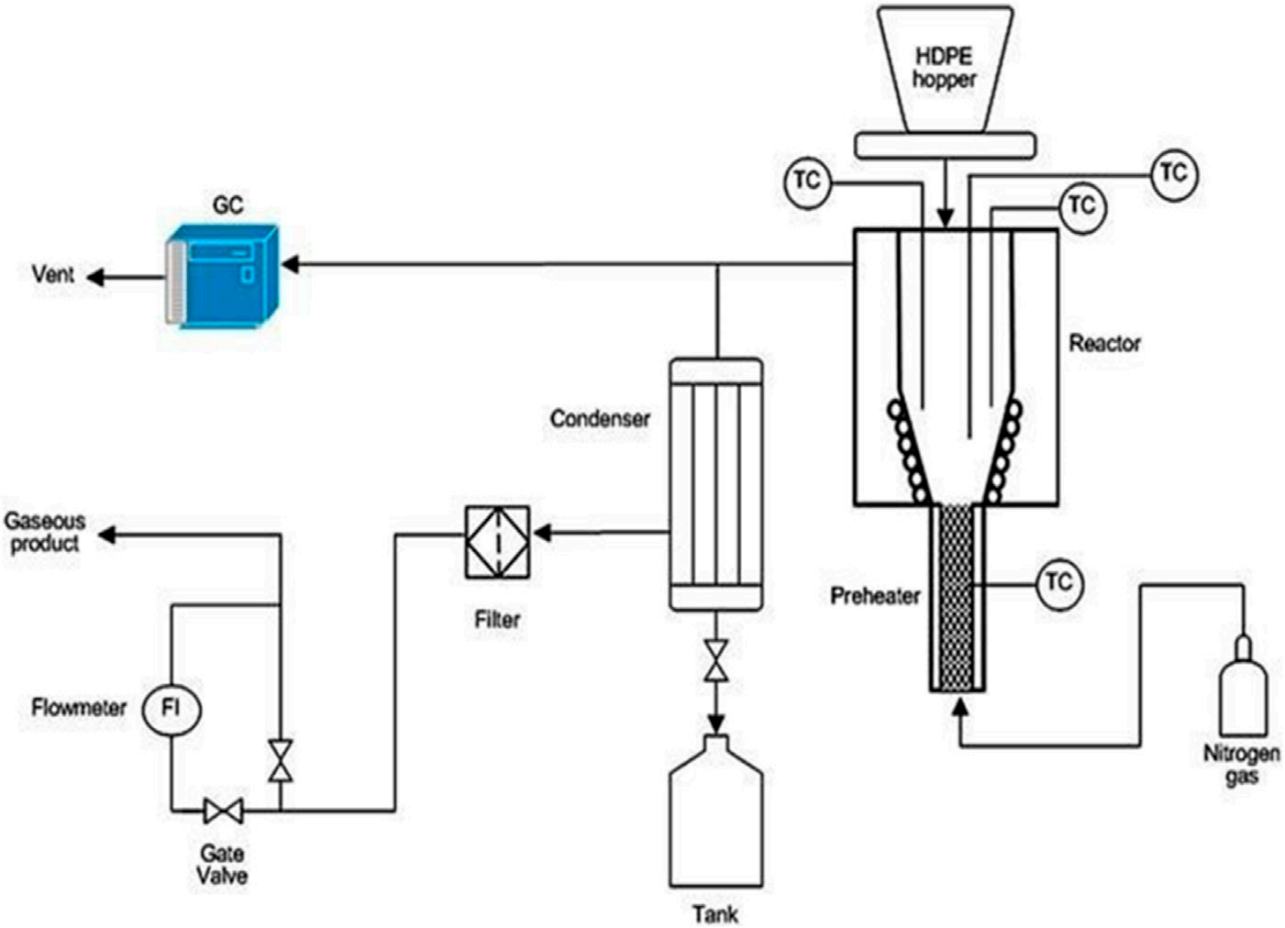
CSBR illustration in pyrolysis of HDPE in the presence of zeolite catalyst (Elordi et al., 2007).

Elordi et al. (2007) studied the pyrolysis of HDPE illustrated in Figure 13 at 500 °C in the CSBR by utilizing HY zeolite catalyst. The gasoline fraction yield was 68.6 wt% (C5–C10). The octane number of the gasoline was RON 96.6, which is similar to the conventional gasoline quality. Arabiourrutia et al. (2012b) utilized the CSBR to investigate the depiction and wax yield from the pyrolysis processes of PP, LDPE, and HDPE at 450–600 C. They claimed that the CSBR has the ability to handle sticky solids that are difficult to handle in the FBR. The spouted bed scheme was specifically well suited to low-temperature wax pyrolysis. They found that with the temperature, the yield of waxes decreased. More wax is cracked into gaseous and liquid products at higher temperatures. The yield of waxes from the PP pyrolysis was 92 wt%, while that from LDPE and HDPE was very similar at 80 wt% waxes.

Artetxe et al. (2015) studied the flash pyrolysis of PS in the CSBR for styrene recovery at 450–600°C. The results showed that gas velocity and temperature have a significant impact on the yield of styrene, having maximum recovery of the monomer (70.6 wt%) at 500°C. Regarding light olefins recovery, the same authors performed a two-step pyrolysis process in CSBR. The light olefins yield was 77 wt% in the second step at 900°C. Moreover, the yield of butene, propylene, and ethylene was 17.5%, 19.5%, and 40.4% respectively. On the other hand, the yield of aromatics was only 6.2 wt% (Artetxe et al., 2012; Barbarias et al., 2018). These results show that the CSBR enables optimizing wax yield and preventing problems of defluidization.

### 9.4 Microwave-assisted technology

Microwave heating is used in microwave pyrolysis, and it now provides a novel method for the recovery of waste through the process of pyrolysis. In this method, waste materials are mixed with a microwave-absorbent substance like particulate carbon. The microwave energy is absorbed by the microwave absorbent to generate enough thermal energy to reach the temperatures required for comprehensive pyrolysis (Lam and Chase, 2012). Microwave pyrolysis has a number of benefits over the traditional pyrolysis process, such as lower particle levels in bio-oils, ease of control, and standardized large biomass internal heating. In this process, the material is directly heated with microwave energy that is directly delivered by using molecular interactions with the electron beam (Fernandez et al., 2011). This would heat up the environment without wasting any time. Regardless of the benefits of microwave heating, there is a significant drawback that prevents this technique from being extensively investigated on the commercial scale, such as the lack of adequate evidence to measure the treated waste stream’s dielectric properties. Microwave heating efficiency is highly dependent on the material’s dielectric properties. Plastics, for example, have a low relative permittivity, so during pyrolysis, combining them with a microwave absorber (carbon) can allow more energy to be absorbed and altered into heat in less time (Lam and Chase, 2012). As a result, the heating efficacy of each material can vary, posing a significant problem to the industries.

Ludlow-Palafox and Chase (2001) studied microwave-assisted pyrolysis by using two different substances: i) HDPE small pallets and ii) toothpaste packaging combined with polyethylene laminates and aluminum. This experiment is unique in that it includes a 180-cm diameter quartz vessel reactor with a 6-RPM impeller that mounted within the microwave. With a microwave power of 5 kW, carbon is used as the absorber. The liquid oil yield from the pyrolysis of HDPE was 79–81 wt%, with a gaseous yield of 19–21 wt% and no solid residue at 500–600°C. On the other hand, no product was produced from polyethylene laminates and aluminum pyrolysis. Moreover, the authors have noted that at the same operating temperatures, there was no discernible variance in product yield between the HDPE pellets and laminates. The average molar mass was somewhat higher in both cases, but the molar mass distribution was corresponding. Since aluminum was easily removed by sieving, it had no effect on the product yield. They noticed a substance called titanium dioxide (TiO_2)_ adhered to the side walls of the reactor as a white powder during the experiment. TiO_2_ can be seen on the toothpaste tube’s painted surface. Since it had segregated from the laminate’s organic content during pyrolysis, this material had no effect on the yield of the pyrolysis product. Conclusively, using the microwave-assisted pyrolysis process, real waste like toothpaste packaging was pyrolyzed successfully.

Undri et al. (2014) also studied the microwave heating technology in the pyrolysis process by utilizing two sorts of absorbers (carbon and tires). The waste plastics used were HDPE and PP (polyolefin). Microwave power ranging from 1.2 to 6.0 kW was used. They obtained 74.8 wt% liquid yield from PP, whereas they found the highest yield of 90 wt% from HDPE. Carbon was used as the microwave absorber in both experiments, with microwave powers varying from 3 to 6 kW because the polymers’ residence time in the oven was shortened by using high power. As a result, instead of non-condensable gases, more polymers were transfigured to liquid. The solid residue level increased to 33 wt% when tires were used as the microwave absorber, which was due to other non-pyrolyzable compounds in the tires. Due to the cocking phase, the accumulated solid residue was at its lowest at 0.4 wt% when compared to when using carbon as the microwave absorber. Carbon was found to be a strong microwave absorbent, with a high capability for converting and absorbing microwave energy into heat. In order to optimize the yield of liquid in microwave pyrolysis, special attention must be given to the absorber type and microwave power.

Khaghanikavkani (2013) also studied microwave technology and elaborated on multiple variables that effect the performance of microwave heating in the pyrolysis of plastic such as the design of microwave rotation, absorber type, and nitrogen volume velocity. Lam and Chase (2012), Fernandez et al. (2011), and Undri et al. (2011) have also published comprehensive analyses of the microwave heating technology in the plastic pyrolysis.

## 10 Process parameters that influence pyrolysis process

In any process, the parameters play an important role in the optimization of the product composition and yield. The yield of the final end products, for instance, char, liquid oil, and gas, can be influenced by the main process parameters in plastic pyrolysis. Monitoring the parameters at various settings will result in the required product. These parameters are elaborated on in the following section.

### 10.1 Residence time and pressure

Residence time is one of the key parameters that influences the end product yield and is defined as the amount of time (average) that a particle or substance takes in the reactor (Mastral et al., 2001). Prolonged residence time improves the primary products' conversion, resulting in more thermally persistent products like non-condensable gas and lower-molecular-weight compounds (Ludlow-Palafox and Chase, 2001). In the fluidized bed reactor, Mastral et al. (2003) explored the temperature effect and residence time on the HDPE pyrolysis product distribution. They discovered that high residence time yields a higher liquid product when the temperature does not exceed 685°C. At temperatures above 685°C, however, the influence of residence time is less on the yield of the gaseous and liquid products.

Murata et al. (2004) investigated the effect of pressure in a continuous stirred tank reactor on the HDPE pyrolysis at 0.1–0.8 MPa at an elevated temperature. They found that as the pressure increased from 0.1 to 0.8 MPa, the gaseous product yield increased dramatically from about 6 wt% to 13 wt% at 410°C, but only slightly at 440°C from 4 wt% to 6 wt%. This demonstrates that at elevated temperatures, pressure had a significant effect on the gaseous product distribution. At a high pressure, the liquid product carbon number distribution shifted to the smaller molecular weight side. Murata et al. (2004) discovered that when the pressure was increased, a decrease in the double bond formation occurs. This indicated that the C–C links scission rate in polymers was directly influenced by pressure, suggesting that pressure had a major impact on the rate of formation of double bonds. They also found out that at lower temperatures, pressure had a significant effect on residence time. As the temperature exceeded 430°C, however, the pressure effect on the residence time became less noticeable.

As a result, it was concluded that both residence time and pressure are temperature-dependent variables that at lower temperatures, may affect the product distribution of plastic pyrolysis. The yield of gaseous products improved at higher pressures and influenced the gaseous and liquid products' molecular weight distribution, but only at very high temperatures. The residence time effect at higher temperatures becomes less evident, which is why most of the researchers focus more on the temperature parameter rather than on residence time while conducting plastic waste pyrolysis studies. Furthermore, if the pressure factor is deemed, additional units like pressure transmitter and compressor must be augmented to the entire system, thus increasing the operating cost.

### 10.2 Temperature

In the pyrolysis process, temperature is one of the most important and key variables because it controls the polymer chain’s cracking process. The molecules are prevented from collapsing by the Van der Waals force which attracts them together. In the system, the molecules' vibration increases when the system temperature rises, thus causing the molecules to evaporate from the system surface. When the energy impelled by the intermolecular force along the polymer chains exceeds the C–C bond enthalpy in the chain, the carbon chain breaks (Sobko, 2008). The thermogravimetry analyzer is used to measure the plastics' thermal cracking behavior. The thermogravimetry analysis (TG) curve and derivative thermogravimetry analysis (DTG) curve are two types of graphs produced by the analyzer. The TG curve calculates a substance’s weight change as a function of temperature and time, while the DTG curve provides data on the degrading phase that occurs through the process, as shown by the number of peaks (Kumar and Singh, 2011). Cepeliogullar et al. (2013) studied the pyrolysis of PET in which they observed that at a temperature of 427.8°C, the material’s maximum weight loss occurred. At 400°C, actual degradation of PET started and when the temperature was between 200 and 400°C, small changes in weight loss occurred. They also noted that above 470°C, there were no considerable alterations. Hence, the temperature range of the PET thermal degradation is 350–520°C.

Cepeliogullar et al. (2013) studied the thermal behavior of PVC and reported two significant weight losses at two distinct temperature variations. The first temperature variation was 260–385°C, which resulted in a peak weight reduction of 62.26% when compared to the starting weight. The second temperature variation was between 385 and 520°C, which resulted in a decrease in weight of 21.76% when compared to the initial weight. The material weight loss became minimal as the temperature was increased to 800°C (1.63%). Thus, the PVC degradation temperature was between 200 and 520°C. Chin et al. (2014) studied the HDPE thermal degradation at the heating rates of 10–50°C/min. Based on the TG analysis, they observed that the thermal degradation of HDPE was completed almost at 517–538°C that began at 377–404°C. The weight loss was accelerated with higher heating rates, which increased the reaction rate. In a subsequent study conducted by Marcilla et al. (2005), the authors observed that at 468°C, the HDPE degradation rate was at its maximum.

Jung et al. (2010) investigated the temperature effect on the pyrolysis processes of HDPE and PP in a fluidized bed reactor and observed that the main HDPE and PP degradation started at 400–500°C according to the DTG curves. However, while comparing the PP and HDPE fractions, it was discovered that the PP fraction had begun losing weight at temperatures lower than 400°C. Marcilla et al. (2005), on the other hand, had found out that for HDPE, the greatest degradation temperature was at 467°C, while PP degraded at 447°C. In principle, the degradation rate of HDPE was slower than that of PP because of its linear structure which contains very little branching that has stronger intermolecular forces (Jung et al., 2010).

Marcilla et al. (2009b) found that at 360–385°C, a small volume of liquid oil was formed during the LDPE pyrolysis. At 469–494°C, the highest liquid yield was obtained. Onwudili et al. (2009) found that below 410°C, a brown waxy substance was produced, and at 410°C, the actual LDPE oil conversion had started. They also observed that at 425°C, the highest liquid yield was obtained. Marcilla et al. (2009a) had also observed the highest liquid oil yield at 550°C from LDPE degradation. Increasing the temperature to 600°C did not improve the liquid oil yield (Williams and Williams, 1998). Therefore, the optimum temperature for liquid oil production from LDPE is 360–550°C. Onwudili et al. (2009) explored the pyrolysis of PS in a batch reactor. At 350°C, they obtained highly viscous dark-colored oil, and no PS degradation occurred at 300 C. At 425°C, the highest yield of liquid oil was achieved. Higher temperatures up to 580°C increased the production of gaseous products while lowering the liquid oil yield (Demirbas, 2004). Therefore, PS thermal cracking temperature is estimated to be between 350 and 500°C.

As a result of the previous discussion, it has been established that the reaction rate depends mainly on temperature which greatly affects the output composition for all plastics. The product selectivity depends strongly on the operating temperature. Lower temperatures of 300–500°C yield liquid products while a higher temperature of 500°C or more yields char or gaseous products.

### 10.3 Catalysts

Catalysis refers to a substance’s ability to accelerate the rate at which a chemical reaction occurs. Catalysts help to make chemical production faster, easier, and safer. They do so by controlling the activation energy necessary to initiate chemical reactions. Because heat is the most costly factor in industries, using a catalyst for it could help save energy. As a result, catalytic degradation is especially appealing for obtaining commercially valuable products such as C2–C4 olefins and automobile fuel (gasoline and diesel) which are in high demand in the petrochemical industry (Elordi et al., 2009). Furthermore, many studies have employed catalysts for product enhancement to increase hydrocarbon distribution to generate pyrolysis liquid with qualities comparable to traditional fuels such as diesel and gasoline.

#### 10.3.1 Zeolite-based catalysts

Zeolites are basically crystalline aluminosilicate (Al_2_SiO_5_) sieves with open pores that have ion exchange properties (Degnan, 2000; International Zeolite Association, 2005). The framework is made up of a three-dimensional structure that connects the tetrahedral sides with oxygen atoms. Zeolite catalyst is build by various SiO_2_/Al_2_O_3_ ratio which depends largely on its type. The reactivity of zeolite is determined by the SiO_2_/Al_2_O_3_ ratio, which influences the pyrolysis end product. The aromatics and light alkanes yield is also decreased when zeolite’s SiO_2_/Al_2_O_3_ ratio is increased. Table 7 equates the gasoline fraction fuel qualities achieved with three types of HZSM-5 zeolite with varying SiO_2_/Al_2_O_3_ ratios. As shown, the lowest catalyst acidity with the highest SiO_2_/Al_2_O_3_ ratio resulted in higher olefin content but lower benzene and aromatics content and lower octane number. In the pyrolysis of HDPE, HZSM-5 zeolite’s SiO_2_/Al_2_O_3_ ratio had a considerable impact on the yield of product fraction (Artetxe et al., 2013b). The zeolite’s low acidity was signified by a high SiO_2_/Al_2_O_3_ ratio. When compared to the highly acidic catalyst (SiO_2_/Al_2_O_3_ = 30), the low acidic catalyst (SiO_2_/Al_2_O_3_ = 280) was less dynamic in breaking waxes, resulting in higher C12–C20 fractions and lower light olefins. The yield of light olefins decreased from 58.0 to 35.5 wt% when the SiO_2_/Al_2_O_3_ ratio was increased from 30 to 280, but the yield of C12–C20 fraction had increased from 5.3 wt% to 28.0 wt%. Marcilla et al. (2008) investigated the performance of HZSM-5 and HUSY in a batch reactor at 550 °C with a 10 wt% polymer-to-catalyst ratio on HDPE and LDPE. The HZSM-5 catalyst produced more gaseous product (LDPE = 70.7 wt%, HDPE = 72.6 wt%). Conversely, when compared to the HZSM-5 catalyst (LDPE = 18.3 wt%, HDPE = 17.3 wt%), more liquid oil was obtained with the HUSY catalyst (LDPE = 61.6 wt%, HDPE = 41.0 wt%). Lin and Yen (2005) showed a similar tendency of product selectivity utilizing HUSY and HZSM-5 zeolites on the pyrolysis of PP. This demonstrates that product selectivity varies depending on the catalyst and different zeolite catalysts may have distinct product predilection in terms of selectivity. In the catalytic pyrolysis of plastics, HMOR and HUSY are extensively utilized zeolite catalysts. Garfoth et al. (1998) examined the efficacy of three zeolite catalysts for HDPE pyrolysis: HUSY, HMOR, and HZSM-5 with a 40 wt% polymer-to-catalyst (P/C) ratio. In their experimentation, they observed that the catalytic activity of HUSY and HMOR was less when compared to that of HZSM-5. HUSY and HMOR left 7.08 wt% and 8.94 wt% residues, while HZSM-5 left 4.53 wt% residue which indicates higher catalytic capabilities of HZSM-5 over HUSY and HMOR.

**TABLE 7:** Comparison of gasoline fraction fuel properties achieved with various SiO_2_/Al_2_O_3_ ratios using three HZSM-5 types (Artetxe et al., 2013b).

SiO_2_/Al_2_O_3_	Aromatics (vol%)	Olefins (vol%)	Benzene (vol%)	Octane number
280	6.9	68.9	0.46	85.9
80	13.5	62.2	1.3	86.7
30	43.3	33.1	4.2	94.1
Required	<35	<18	<1	95

Miskolczi et al. (2009) studied the effect of zeolite catalyst in real municipal plastic waste pyrolysis. PP and HDPE waste sources were collected from packaging agriculture sectors, and before pyrolysis, they were chopped and washed. Both polymers (PP = 35 mg kg^−1^ and HDPE = 238 mg kg^−1^) contain sulfur according to the properties analysis, although more contaminants were found in the HDPE waste generated from agricultural sectors such as calcium (103 mg kg^−1^), phosphorus (47 mg kg^−1^), and nitrogen (963 mg kg^−1^). The contaminants were most likely caused by fertilizer containing superphosphate and ammonium nitrate, which could have collected in the HDPE waste after the washing method failed to remove them. With 40 wt% HZSM-5 catalyst, the catalytic pyrolysis was performed at 520°C. The utilized structure of the catalyst was analyzed by EDAX and SEM after the pyrolysis. Aside from the silica–alumina HZSM-5 zeolite structure, traces of phosphorus, nitrogen, and sulfur were found on the catalyst surface (sodium, oxygen, magnesium, aluminum, calcium, silica, and potassium). This shows that the contaminants were derived from plastic waste. However, the product characteristics were not affected by the catalyst surface contaminants, which were influenced more by the catalytic pore structure and grain diameter. In fact, the plastic waste pyrolysis catalyst may be reused because the pore diameter is determined to be the same as that of the new catalyst (Miskolczi et al., 2009). In the pyrolysis of HDPE waste, sulfur content was reduced dramatically from 75 mg kg^−1^–37 mg kg^−1^ when the HZSM-5 catalyst was used which shows that the catalyst usage facilitates minimizing contaminants in the oil. The phosphorus and nitrogen content showed a similar reduction pattern. Calcium content was exclusively found in heavy fuel oil fraction, while no calcium was found in the light oil or gasoline fractions.

Seo et al. (2003) studied the HZSM-5 effect at 450°C in the pyrolysis of HDPE. They found that HZSM-5 produced a higher gaseous yield (63.6 wt%) but very low liquid product (35 wt%) with a 20 wt% catalyst-to-polymer ratio. Hernández et al. (2007) acquired a high gaseous yield (86.2 wt%) but lower liquid product (4.4 wt%) than did Seo et al. (2003) at 500 C. Lin and Yen (2005) obtained very low 2.31 wt% and 3.75 wt% liquid yield at 360 C in PP pyrolysis by utilizing HZSM-5 and HUSY zeolites with a 40 wt% catalyst-to-polymer ratio. However, the coking resistance of HZSM-5 was higher than that for HUSY when the product stream such as pentene and butane increased during the process while iso-pentanes and iso-butane persisted unaffected (Uemichi et al., 1998; Lin and Yen, 2005; Obeid et al., 2014).

Apart from direct plastic cracking, some authors have also studied the effectiveness of zeolite catalysts in two-step reaction processes incorporating catalytic and thermal reactors (Vasile et al., 2000; Syamsiro et al., 2014). In the two-step reaction process, Aguado et al. (2007) investigated the LDPE catalytic conversion in a batch reactor and fixed-bed reactor. In the batch reactor, the plastic would be thermally cracked, and in the fixed-bed reactor, the generated vapors were carried out where the catalyst HZSM-5 (10 wt%) was placed. Pyrolysis was carried out at a temperature of 425–475°C. Catalytic reforming with a zeolite catalyst increased the gas fraction significantly which was around 74.4 wt%, however, the liquid oil yield was only 22.0 wt% at the maximum temperature. As a result, the observed trend was extremely corresponding to catalytic direct degradation, which yielded a high gaseous product when the HZSM-5 catalyst was used.

#### 10.3.2 Fluid catalytic cracking

Fluid catalytic cracking (FFC) catalysts are commonly employed in oil refinery processes to break the chains of high-molecular-weight hydrocarbons, which is required to maximize the amount of gasoline produced. Nowadays, FCC catalysts are made up of zeolitic materials and different promoters and binders (Humphries and Wilcox, 1989; Rajagopalan and Habib, 1992; Magee and Mitchell, 1993; Degnan, 2000). Due to its strong thermal stability and product selectivity, Zeolite-Y has been the major FCC catalyst component for over 40 years (Marcilly, 2000). Kyong et al. (2002) studied the spent FCC catalyst effect at 400°C on LDPE, HDPE, PS, and PP pyrolysis in a stirred semi-batch reactor at 7°C/min heating rate. They found that PS produced 90 wt% liquid yield which was the highest among all other plastics (others produced over 80 wt%). In terms of the gaseous product yield, PE produced the highest gaseous yield followed by PP and PS. The liquid product yields which had an opposing order with PS produced the highest followed by PP and PE (LDPE, HDPE). PS produced less gaseous product because it had a benzene ring that formed a more firm structure. Overall, spent FCC catalyst exhibits good catalytic efficacy, with liquid yields over 80% for all plastic specimens. Furthermore, because it is a “reused” catalyst, it is less expensive.

Using the same experimental conditions, Kyong et al. (2003) studied the spent FCC catalyst's efficiency in comparison to HDPE thermal pyrolysis without a catalyst, but with a temperature of 430°C. They observed that with the catalyst, the gaseous yield slightly reduced from 20.0 to 19.5 wt% while increasing the yield of liquid oil from 75.6 to 79.8 wt%. The presence of the catalyst also reduced the solid residue from 4.5 to 0.8 wt%. Furthermore, the formation of liquid oil from HDPE was observed at 350°C, which means that the FCC catalyst lowered the HDPE reaction's temperature. In the case of thermal pyrolysis, the initial liquid formed at 430 °C after 30 min. This means that in thermal pyrolysis, using the spent FCC catalyst improved the overall product conversion while also increasing the reaction rate.

Apart from this, the plastic pyrolysis product distribution may be affected by different FCC conditions. FCC steaming catalyst, for example, would change the composition and structure of the catalyst. Olazar et al. (2009) proved this by conducting a study on severe, mild, and fresh FCC catalyst steaming. Severe steaming was performed for 8 h at 816 °C, while mild steaming was performed for 5 h at 760°C. The results exhibited that steaming increased the FCC catalytic performance. As shown in Table 8, the fresh FCC catalyst yields a high gaseous fraction and low diesel fraction, while severe FCC steaming produced less gaseous products (C1–C4 hydrocarbon) and a high diesel fraction (C10 + hydrocarbon).

**TABLE 8:** FCC fresh and steaming product distribution (Olazar et al., 2009).

FFC catalyst type	Medium gasoline (C5–C9) (wt%)	Diesel (C10+) (wt%)	Gaseous (C1–C4) (wt%)
Severe steaming	20	70	5
Mild steaming	38	40	25
Fresh FCC	35	15	52

Besides this, the polymer to catalyst ratio also greatly affects the product composition and yield. Abbas-Abadi et al. (2014) investigated the various HDPE to FCC catalyst ratios ranging from 10 to 60 wt% at 450°C in a stirred semi-batch reactor. They observed that the catalyst to polymer ratio of 20 wt% was the prime ratio for higher liquid yield conversion. The coke obtained was around 4.1 wt% with a very high liquid yield of 91.2 wt% and gaseous product of 4.7 wt%. A decrease in liquid production was observed by increasing more than 20 wt% of the catalyst to polymer ratio, thus producing high gaseous product and coke. This indicates that the catalyst/polymer ratio had to be constrained in order to improve the conversion of the product, particularly the yield of liquid oil and catalyst coke formation. Kyong et al. (2002) explored the effectiveness of the FCC catalyst with a catalyst to polymer ratio of 10 wt% to the various plastics types. The results showed that a high yield of liquid was produced (80–90 wt%) for PS, PP, HDPE, and LDPE, which shows the productivity of the FCC catalyst in the pyrolysis of different plastic waste. Similarly, Rodríguez et al. (2019) studied the pyrolysis of HDPE waxes using the FCC catalyst at 3, 5, and 7 g_cat_ g_oil_^−1^ ratio at 500–600°C and obtained HDPE waxes product distribution of 36.7–65.1 wt%. Furthermore, Palos et al. (2022b) under similar conditions used three FCC catalysts and obtained 82.0 wt% heavy cycle oil and 12.5 wt% light cycle oil. Moreover, Abbas-Abadi et al. (2014) also achieved a very high yield of liquid oil (92.3 wt%) at 450 °C in PP pyrolysis with a 10 wt% catalyst/polymer ratio. Conclusively, the utilization of the FCC catalyst was recommended in the pyrolysis of plastic to optimize the production of liquid oil. But in order to obviate the gaseous product and coke dominance, the catalyst/polymer ratio must not exceed 20 wt%.

#### 10.3.3 Silica–alumina catalyst

The amorphous acidic catalyst silica–alumina has Bronsted acid sites with ionizable hydrogen atoms and Lewis acid sites which accept electrons. The SiO_2_/Al_2_O_3_ molar ratio determines the acid content of silica–alumina catalyst. Opposite to zeolite, a high SiO_2_/Al_2_O_3_ ratio means high silica–alumina catalyst acidic strength. For example, SiO_2_/Al_2_O_3_ = 0.27 (SA-2) has a low acidic strength than SiO_2_/Al_2_O_3_ = 4.99 (SA-1), and both are commercial silica–alumina (Sakata et al., 1997). The catalyst mode also plays an important role in product distribution and the product yield. This was proven by Sakata et al. (1997) on the pyrolysis of PP at 380°C by utilizing the silica–alumina catalyst with different contact modes: vapor phase and liquid phase. The catalyst was assorted with PP pallets and in the liquid phase was placed into the batch reactor. In contrast to the vapor phase, the catalyst was suspended 10 cm from the reactor’s bottom on a stainless steel net. From the experiment, they observed that a higher gaseous product (35 wt%) was produced when the catalyst was in the vapor phase and a low liquid product was produced because over the silica–alumina catalyst, the polymer decomposed further into the gaseous product. Alternatively, the catalyst in the liquid phase produced little gaseous product but a higher yield of liquid (68.8 wt%) because the wax residue over the silica–alumina catalyst disintegrated into a lighter hydrocarbon.

In plastic pyrolysis, the final end product is greatly influenced by the catalyst's acidic strength. At 430°C, Sakata et al. (1997) investigated catalysts' acidity effect on HDPE pyrolysis product distribution in semi-batch reactor where 10 g of HDPE was mixed with 1 g of the catalyst (SA-1, SA-2, ZSM-5). The catalysts' acidic strength was determined by using TPD (NH_3_ temperature programmed desorption). From the results, it was shown that SA-1 had high acidity followed by ZSM-5 and SA-2. From the experimentation, they found the following liquid oil yield order: ZSM-5 (49.8 wt%) < SA-1 (67.8 wt%) < SA-2 (74.3 wt%). The catalyst with lower acidity (SA-2) produced high liquid oil, while ZSM-5 having strong acidic sites produced a low liquid yield when compared to the other catalysts but produced high gaseous product. Uddin et al. (1996), by using the same experimental conditions as Sakata et al. (1997), also investigated the SA-2 effect on LDPE and HDPE pyrolysis processes and obtained high liquid oil by using LDPE (80.2 wt%) than using HDPE (77.4 wt%). The LDPE structure was weaker than that of HDPE because of its branched chain, thus LDPE produced high amounts of liquid yield. Moreover, the catalyst reactivity can also be augmented under specific temperature ranges. Luo et al. (2000) studied PP and HDPE pyrolysis processes in a fluidized bed reactor at 500 °C by utilizing a silica–alumina catalyst, and they obtained higher liquid oil than did Sakata et al. (1999) and Uddin et al. (1996). The liquid product obtained was 90 wt% for PP pyrolysis and around 85.0 wt% for HDPE pyrolysis. This demonstrates that temperature is also crucial in maximizing catalyst effectiveness in the process of plastic pyrolysis to optimize the yield of the liquid oil product. In conclusion, the FCC catalyst is the ideal catalyst in plastic pyrolysis for optimizing liquid oil production. The FCC catalyst in the pyrolysis processes of PP and HDPE produced 90 wt% liquid oil while the highest yield of liquid for HDPE and PP by using silica–alumina was around 85–87 wt% (Luo et al., 2000; Abbas-Abadi et al., 2013; Abbas-Abadi et al., 2014). This shows the effectiveness of the FCC catalyst in plastic pyrolysis for product optimization and is also more economically attractive than zeolite-based catalysts.

### 10.4 Fluidizing (medium) gas effect in the pyrolysis process

Fluidizing gas (also known as inert gas) is a carrier gas that is only used to carry vaporized products and does not participate in the pyrolysis process. The reactivity of the fluidizing gas (each type) depends on its molar mass. Propylene, ethylene, hydrogen, argon, nitrogen, and helium are some of the fluidizing gases that can be utilized in the pyrolysis of plastics. According to Abbas-Abadi et al. (2014), the carrier gas’s molecular size aids in defining the product composition, which is also affected by temperature. The PP catalytic pyrolysis product distribution was affected by the carrier gas’s molecular weight, as shown in Table 9. High amounts of liquid oil (condensed product) were produced by the lighter gas; 33.8 wt% liquid was produced without using any carrier gas, while 96.7 wt% of the liquid oil was produced by using H_2_ as shown in Table 9. This demonstrates the importance of carrier gas in improving pyrolysis product yield. Apart from this, it has been discovered that the carrier gas’s reactivity influenced the formation of coke. Ar coke formation was very high followed by N_2_, propylene, and helium, while H_2_ coke formation was very low. The molecular weights of nitrogen and ethylene were the same. However, the reactivity of ethylene was higher and produced lower coke and high liquid oil yield than nitrogen because it could cause the equilibrium to shift, resulting in a higher liquid yield (Abbas-Abadi et al., 2014). However, in plastic pyrolysis, propylene and hydrogen were used the least by many researchers because of the flammability risk, while nitrogen was the more commonly utilized fluidizing gas since it is safer and easier to handle.

**TABLE 9:** The effect of carrier gas on the product yield and the condensed product composition (Abbas-Abadi et al., 2014).

Fluidizing gas	Molar mass	Yield of non-condensable product (%)	Yield of condensed product (%)	Olefins (%)	Coke yield (%)	Naphthenes (%)	Paraffins (%)	Olefins/paraffin ratio	Aromatics
Ar	37	9.8	84.8	45.21	5.4	21.93	25.27	0.66	7.59
Propylene	42	9.7	87.8	42.36	2.5	20.92	31.85	1.33	4.87
Ethylene	28	5.1	93.8	41.76	1.1	19.75	34.76	1.2	3.73
N_2_	28	4.1	92.3	44.63	3.6	17.23	32.87	1.36	5.27
He	4	3.2	94.7	43.32	2.1	19.29	33.41	1.3	3.98
H_2_	2	3	96.7	30.86	0.3	20.54	46.53	0.66	2.07

Besides this, the flow rate of the fluidizing gas may also affect the final end product. This was proven by Lin and Yen (2005) by using the HUSY catalyst over PP pyrolysis at 360 °C. They observed that at 300 ml/min (the lowest fluidizing flow rate), the degradation rate decreased instantly. At a lower flow rate, the primary product contact time was high, leading the coke precursor formation to enhance with the by-product achieved despite the rate of degradation being slower (Lin and Yang, 2007). At 900 ml/min (highest fluidizing flowrate), the hydrocarbon gases and gasoline fractions were increased. As a result, in plastic pyrolysis, the rate and type of fluidizing gas are particularly important, as they certainly affect the composition of the end product.

## 11 Pyrolysis and in-line steam reforming

Because of the high production of H_2_ and operational dominance, the two-step pyrolysis and in-line catalytic reforming of plastic waste (Table 10) is likely the most propitious (Wu and Williams, 2009a; Wu and Williams, 2010a; Namioka et al., 2011; Barbarias et al., 2016b; Arregi et al., 2017). Moreover, waste plastic contaminants remain in the reactor, avoiding contact which resulting catalyst deactivation (Wu and Williams, 2010c). The steam catalytic reforming and thermal degradation steps may potentially benefit from independent temperature maximization (Park et al., 2010). In addition, when compared to direct gasification, the process temperature is substantially lower, reducing reforming catalyst sintering issues and material costs (Barbarias et al., 2016a; Barbarias et al., 2016b). As a result, this process removes tars completely from the gaseous product because of the usage of the very active reforming catalyst, which is the main advantage of this process. This approach has also been shown to be useful in H_2_ production from biomass (Xiao et al., 2013; Ma et al., 2014; Arregi et al., 2016). Furthermore, a new option for the plastic waste pyrolysis–reforming method has recently been presented, which entails producing carbon nanotubes and H_2_, simultaneously utilizing various Fe- and Ni-based catalysts (Yang et al., 2015; YangRChuang and Wey, 2016; Bajad et al., 2017; Liu et al., 2017; Yao et al., 2017).

**TABLE 10:** Results obtained by different authors by using pyrolysis and steam reforming technique.

Reactor	Approach	Plastic type	Reaction Conditions (°C, -)	Bed material	Composition of gas (% vol)	Gas yield (m^3^/kg)	Production of H_2_ (100g/plastic)	Tar yield (g /m^3^)	References
Plasma reactor (11 kg h^−1^)	Plasma gasification with CO_2_	PE, PET and PP mixture	T: 1200–1400	-	CO: 50, H_2_: 42, CH_4_: 0, CO^2^: 7	-	-	< 0.001	Hlina et al. (2014)
Dual fixed bed (1 g)	Pyrolysis–dry reforming of plastic	PE	T: 500/800	-/Ni-Co-Al	-	-	15	0	Saad and Williams, (2016)
Dual fixed bed (1 g)	Pyrolysis–dry reforming of plastic	PP	T: 500/800	-/Ni-Co-Al	-	-	13.6	0	Saad and Williams, (2016)
Dual fixed bed (1 g)	Pyrolysis–dry reforming of plastic	PS	T: 500/800	-/Ni-Co-Al	-	-	7.6	0	Saad and Williams, (2016)
Dual fixed bed (1 g)	Pyrolysis–dry reforming of plastic	PET	T: 500/800	-/Ni-Co-Al	-	-	2.5	0	Saad and Williams, (2016)
Fluidized bed (heterogeneous) (0.08 kg h^−1^)	Plastic pyrolytic oil Steam reforming	Pyrolysis oil of PE	S/C: 3.5(molar), T: 570–800	Ni-Al_2_O_3_	CO: 7–18, H_2_:70, CH_4_:<1, CO_2_: 19–12	4.7–5.8	37	-	Tsuji and Hatayama, (2009)
Fluidized bed (heterogeneous) (0.08 kg h^−1^)	Plastic pyrolytic oil Steam reforming	Pyrolysis oil of PE	S/C: 3.5(molar), T: 600–800	Ni-Al_2_O_3_	CO: 8–16, H_2_: 68, CH_4_:< 1, CO_2_: 20–18	4.6–5.2	31.5	-	Tsuji and Hatayama, (2009)
Two Fixed (packed) bed (0.06 kg/h)	Pyrolysis– in line steam reforming	PP	S/C: 3.6(molar), T:400/580–680	-/Ru-Al_2_O_3_	CO: 9–11, H_2_:71–70, CH_4_: 1.5–1.4, CO_2_: 19–16	5.4–8.8	36.5	0	Park et al. (2010)
Two Fixed (packed) bed (0.06 kg/h)	Pyrolysis– in line steam reforming	PP	S/C: 3.6 (molar), T:400-600/630	-/Ru-Al_2_O_3_	CO: 9–8, H_2_:71–72, CH_4_: 1.5–0.9, CO_2_: 19	5.4–5.6	36	0	Park et al. (2010)
Two Fixed (packed) bed (0.06 kg/h)	Pyrolysis– in line steam reforming	PS	S/C: 3.7(molar), T:400/580–680	-/Ru-Al_2_O_3_	CO: 5–10, H_2_:69–68, CH_4_: 0, CO_2_: 25–21	4.2–5.2	33	0	Namioka et al. (2011)
Spouted (conical) bed/packed bed (fixed) (0.05 kg/h)	Pyrolysis– in line steam reforming	PE	S/C: 3.1, T: 500/700	sand/Ni-CaAl_2_O_4_	CO: 11, H_2_:71, CH_4_:<1, CO_2_: 17	5.4	34.5	0.11	Erkiaga et al. (2015)
Spouted (conical) bed/FBR (heterogeneous) (0.05 kg/h)	Pyrolysis– in line steam reforming	PE	S/C: 3.1, T:500/600–700	sand/Ni-CaAl_2_O_4_	CO: 11, H_2_:71, CH_4_:<1, CO_2_: 17	5.4	37.3	0	Barbarias et al. (2016a)
Spouted (conical) bed/FBR (heterogeneous) (0.05 kg/h)	Pyrolysis– in line steam reforming	PS	S/C: 2.89, T: 500/700	sand/Ni-CaAl_2_O_4_	CO: 14, H_2_: 65, CH_4_: < 0.1, CO_2_: 21	5	29.1	0	Barbarias et al. (2016b)
FBR/FBR (0.06 kg/h)	Pyrolysis– in line steam reforming	PP	S/C: 4.6 (molar), T: 650/850	sand/ commercial Ni catalyst	CO: 12, H_2_: 71, CH_4_: 1.2, CO_2_: 16	5.4	34	0	Czernik and French (2006)
FBR/FBR (0.06 kg/h)	Pyrolysis– in line steam reforming	PP	S/C: 4.6 (molar), T: 650/850, ER: 0.25	sand/ commercial Ni catalyst	CO: 12, H_2_: 65, CH_4_: 1.6, CO_2_: 21	4.1	24	0	Czernik and French (2006)
Dual fixed bed (1 g)	Pyrolysis– in line steam reforming	PP	T: 500/600–900	-/Ni-CeO_2_ ZSM-5	CO: 8–26, H_2_:62–67, CH_4_: 7–4, CO_2_: 16–4	-	27–61	0	Wu and Williams, 2009a
Dual fixed bed (1 g)	Pyrolysis– in line steam reforming	PP	T: 500/600–900	-/Ni-CeO_2_ Al_2_O_3_	CO: 9–27, H_2_:62–65, CH_4_: 4–1, CO_2_: 18–4,	-	13–52	0	Wu and Williams, 2008
Dual fixed bed (1 g)	Pyrolysis– in line steam reforming	PP	T: 500/800	-/Ni-Al_2_O_3_	CO: 20, H_2_:56, CH_4_:6, CO_2_: 9	-	27	0	Wu and Williams, 2009e
Dual fixed bed (1 g)	Pyrolysis– in line steam reforming	PP	T: 500/800	-/Ni-CeO_2_	CO: 6, H_2_:75, CH_4_: 5, CO_2_: 7	-	27	0	Wu and Williams, 2009e
Dual fixed bed (1 g)	Pyrolysis– in line steam reforming	PP	T: 500/800	-/Ni-Mg-Al	CO: 24, H_2_: 64, CH_4_: 1, CO_2_: 10,	4.65	26.6	0	Wu and Williams, 2010c
Dual fixed bed (1 g)	Pyrolysis– in line steam reforming	PS	T: 500/800	-/Ni-Mg-Al	CO: 25, H_2_: 58, CH_4_: 1, CO_2_: 10	3.57	18.5	0	Wu and Williams, 2010c
Dual fixed bed (1 g)	Pyrolysis– in line steam reforming	PE	T: 500/800	-/Ni-Mg-Al	CO: 20, H_2_: 67, CH_4_: 1, CO_2_: 12	3.94	26.0	0	Wu and Williams, 2010c
Dual fixed bed (1 g)	Pyrolysis– in line steam reforming	MSW plastics waste	T: 500/800	-/Ni-Mg-Al	CO: 20, H_2_: 67, CH_4_: 1, CO_2_: 12	3.94	23.6	0	Wu and Williams, 2010c
Conical spouted bed/fluidized bed (0.75g/min)	Pyrolysis– in line steam reforming	MSW plastics waste	T:500/700	-/Ni-Al_2_O_3_ CaAl_2_O_4_	CO: 9.9, H_2_:71 CH_4_: 0, CO_2_: 29.3	-	30.3	0	Barbarias et al. (2018)

Prof. Williams conducted a detail study on the pyrolysis–reforming (in-line) method by using various catalysts on waste plastics (Wu and Williams, 2009b; Wu and Williams, 2009c; Wu and Williams, 2009d; Wu and Williams, 2009e; Wu and Williams, 2010a; Wu and Williams, 2010c; Acomb et al., 2014; Saad et al., 2015b). The experimental setup comprised of dual fixed-bed (packed) reactors operating in batches for the pyrolysis and reforming steps. At 40°C–500°C/min, the volatiles formed in the pyrolysis reactor were subsequently processed in the reforming packed bed reactor (800°C). The production of H_2_ was 26.6 wt% when PP was fed, while PS produced only 18.5 wt% H_2_. In both cases, the Ni-Mg-Al catalyst was used (Cho et al., 2013a). Furthermore, in the reforming of derived PP volatiles, the production of H_2_ was approximately 65%. But this time, the authors used Ni-based commercial catalyst (Wu and Williams, 2008; Wu and Williams, 2009c). The same authors recently used CO_2_ instead of steam to investigate the dry reforming of pyrolysis volatiles from plastics (Saad and Williams, 2016). This innovative approach is an intriguing CO_2_ valorization technique since it achieves nearly complete conversion, with the produced syngas primarily consisting of CO and H_2_. By using PET, PS, PP, and PE at 500 and 800°C, the values of H_2_ production are the following: 2.5, 7.6, 13.6, and 15.0 wt%, respectively. The catalyst utilized was Ni-Mg-Al in the pyrolysis and reforming steps. These results are significantly inferior to those found in pyrolysis and in line with steam reforming.

Czernik and French (2006) studied pyrolysis and in-line steam reforming of PP by using commercial Ni-based catalyst at 650 and 800°C in two FBR reactors. The derived plastic volatiles were completely altered into gaseous stream free of tar, with 34 wt% H_2_ production (34 g 100 g PP^−1^). This yield is 80% of the highest stoichiometrically allowable. By co-feeding air with an equivalence ratio of 0.25 into the reforming step while operating under reforming autothermal parameters, the production of H_2_ was lowered to 24 wt%.

Surprisingly, the process has been run successfully at a steady (equilibrium) state for 10 h without detecting any deactivation of the catalyst. Moreover, plastic pyrolysis and in-line reforming were also investigated by Erkiaga et al. (2015), who developed an experimental unit consisting of a CSBR reactor and fixed-bed (packed) reactor for pyrolysis and the steam catalytic reforming step. HDPE was pyrolyzed at 500 °C and then the reforming step was performed at 700°C using a commercial Ni catalyst. The reforming catalysts showed remarkable efficiency and completely converted the waste plastics into gaseous products, with 34.5 wt% of H_2_ yield, which is 81.6% of the stoichiometric maximum allowable. The formation of coke (4.4 wt% of the feed) is the biggest issue in this process, as it obstructs the flow of the reactant in the reforming fixed-bed (packed) reactor. To avoid these operational and functional challenges, Barbarias et al. (2016a) replaced the fixed-bed reactor with the FBR reactor for the reforming step. During experimentation, they obtained a higher H_2_ yield (38.1 wt%) than did Erkiaga et al. (2015), which accounts for 92.6% allowable stoichiometry. This demonstrates the benefits of employing an FBR reactor for the reforming process. Further research using PS validated the high efficiency of this setup (CSBR and FBR) for the pyrolysis and reforming process (Barbarias et al., 2016b). Namioka et al. (2011) also conducted studies on pyrolysis and in-line reforming, but they used PS instead of HDPE and obtained 29.1 wt% H_2_ yield which was lower than that obtained by Barbarias et al. (2016a). The varying H_2_ concentration of these polymers is related to this result. The deactivation kinetics, as well as the type of the deposited coke, are thus dependent on the hydrocarbons produced during the degradation of the polymer (Barbarias et al., 2016b; Barbarias et al., 2016c).

Park et al. (2010) and Namioka et al. (2011) developed a two-step PP pyrolysis and reforming method based on two fixed-bed (packed) reactors (1 g min^−1^) operating in a continuous framework. The pyrolysis and reforming steps were carried out between 400–600°C and 580–680°C. The reforming step was performed on a Ru-Al_2_O_3_ commercial catalyst. Because of a considerable increase in the yield of coke at high temperatures, the optimal outcomes were achieved at 630°C, which is the average temperature studied. As a result, the hydrocarbon liquids were altered completely into coke and gaseous products at 630 °C and the production of H_2_ reached 34.2 wt%. Using similar experimental conditions and units (Namioka et al., 2011), the same authors investigated the PS two-step pyrolysis and reforming process and obtained a lower H_2_ yield (33.0 wt%) than obtained when using PP.

**FIGURE 14:**
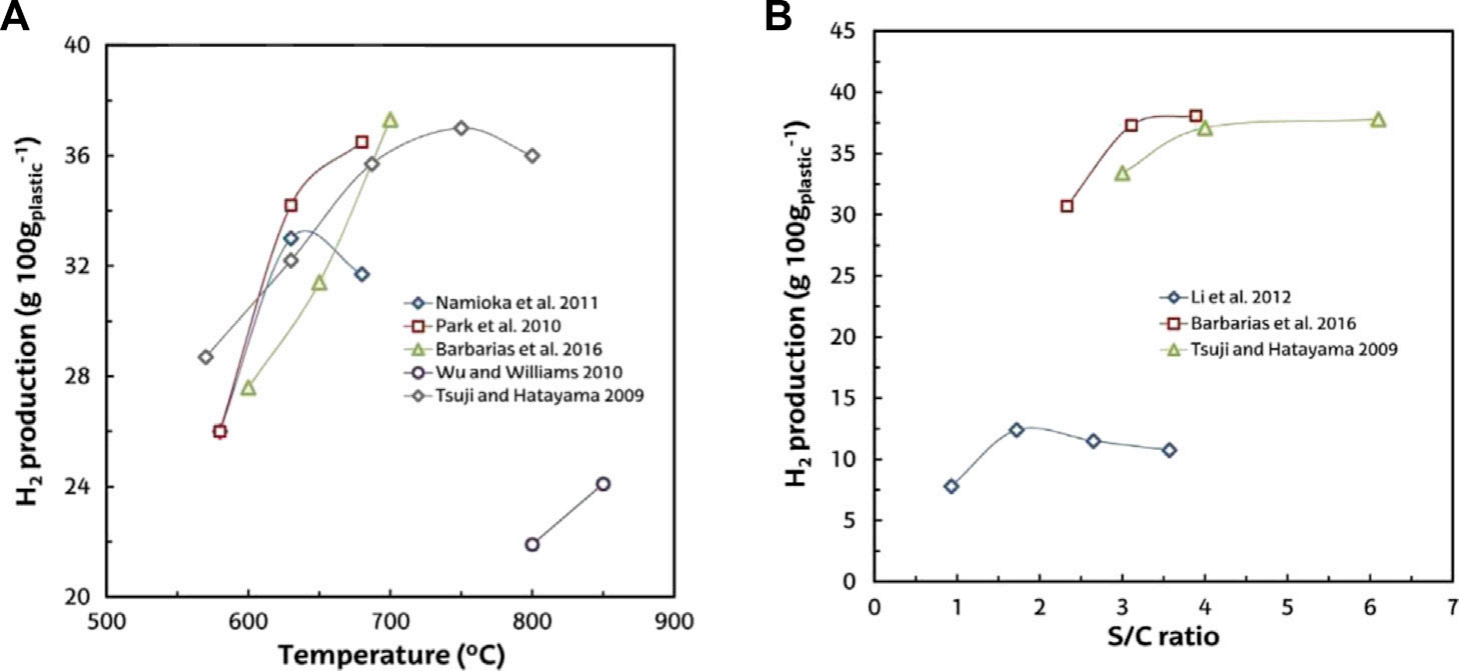
**(A)** Temperature effect on the production of H_2_ in pyrolysis–reforming of plastic. **(B)** Steam/carbon ratio effect on the production of hydrogen in the reforming process of plastic waste.

In comparison to conventional gasification, the pyrolysis two-step and in-line volatiles reforming process allow for 100% conversion, resulting in a gaseous stream with high H_2_ concentration and no tar or liquid hydrocarbons. As a result, different authors have reported the values of H_2_ production above 30 wt% (Tsuji and Hatayama, 2009; Park et al., 2010; Namioka et al., 2011; Erkiaga et al., 2015; Barbarias et al., 2016a; Barbarias et al., 2016b; Arregi et al., 2017). In the pyrolysis–reforming process, the most important parameters which affect the end products are the steam/carbon ratio and reforming phase temperature. Figure 14A depicts the effects of both factors on H_2_ production, respectively. As shown in Figure 14A, the H_2_ production improves by enhancing the reforming temperature, thus increasing the endothermic steam reforming reactions (ESRRs) comprising hydrocarbons, despite the water–gas shift (WGS) reaction equilibrium limiting this improvement. In the reaction environment, the partial steam pressure increases as the steam/carbon ratio rise, enhancing both the water–gas shift reaction and reforming processes, thus favoring the production of H_2_, but at high steam/carbon ratios, this effect is reduced as seen in Figure 14B. However, the indirect approach for the production of H_2_ *via* biomass oil (pyrolysis oil) reforming has been extensively investigated (Trane et al., 2012; Chen et al., 2017; Nabgan et al., 2017), and this route has been studied infrequently in the plastic waste case. Tsuji and Hatayama (2009) only studied the H_2_ production indirect route from plastic waste. The oil produced by the pyrolysis of LDPE was evaporated at 600 and 800 °C and subjected to catalytic steam reforming in a fluidized bed reactor on a Ni-Al_2_O_3_ catalyst. The gas generated has an H_2_ composition of roughly 70% volume, which is near the equilibrium value and contributes to 37.0 wt% of the total production. The oil reforming derived from PS pyrolysis has also been investigated, with the production of H_2_ being 31.6 wt% in this case. Even under ideal conditions, the values of H_2_ production produced in the pyrolysis and in-line reforming approach are substantially greater than those normally achieved in the steam plastics gasification, which are often below 20 wt% (HeMXiao et al., 2009a; Erkiaga et al., 2013a; Martínez-Lera et al., 2013a). Similarly, due to the high H_2_ and carbon content in plastics, the production of H_2_ achieved through biomass pyrolysis–reforming and steam gasification is significantly lower, ranging from 2–8 wt% (Rapagna et al., 2000; Luo et al., 2009; Umeki et al., 2010; Erkiaga et al., 2014) to 4–11 wt% (Xiao et al., 2013; Ma et al., 2014; Arregi et al., 2016). As a result, the pyrolysis–reforming technique for plastic waste valorization is a promising approach.

## 12 Summary of pyrolysis of plastic waste

Table 11 outlines the various parameters which affect the composition of the final end products (gas, liquid, and solid) at different conditions in catalytic and thermal pyrolysis processes. The fluidizing media used in all of the studies was nitrogen gas. Compared with other plastics, PVC and PET generate very low liquid oil yield (based on Table 11), making them less commonly investigated by authors. In pyrolysis, PVC was not recommended since it produces toxic HCL acid and has a low liquid oil yield. Furthermore, the oil produced by PVC includes chlorinated compounds, which potentially decrease the quality of the oil and are also harmful to the environment.

**TABLE 11:** Experimental results of pyrolysis of plastic waste by different authors.

Reactor configuration	Feedstock	Temperature	Heating rate (°C/min)	Pressure	Residence time (min)	Solid (wt%)	Oil (wt%)	Gas (wt%)	References
Batch (sequential)	HDPE	450	—	—	60	19.7	74.5	5.8	Miskolczi et al. (2004)
Batch (sequential)	HDPE	550	5	—	—	0	84.7	16.3	Marcilla et al. (2009a)
Batch (sequential)	LDPE	430	3	—	—	7.5	75.6	8.2	Uddin et al. (1996)
Batch (sequential)	LDPE	550	5	—	—	0	93.1	14.6	Marcilla et al. (2009a)
Batch (sequential)	PP	380	3	1 atm	—	13.3	80.1	6.6	Sakata et al. (1999)
Batch (sequential)	PP	740	—	—	—	1.6	48.8	49.6	Demirbas, (2004)
Batch (sequential)	PS	500	—	—	150	0	96.73	3.27	AdnanShah and Jan, (2014)
Batch (sequential)	PS	581	—	—	—	0.6	89.5	9.9	Demirbas, (2004)
Semi-batch (semi-flow)	HDPE	400	7	1 atm	—	2	82	16	Kyong et al. (2002)
Semi-batch (semi-flow)	HDPE	450	25	1 atm	—	4.7	91.2	4.4	Abbas-Abadi et al. (2013)
Semi-batch (semi-flow)	PP	400	7	1 atm	—	2	85	13	Kyong et al. (2002)
Semi-batch (semi-flow)	PP	450	25	1 atm	—	3.6	92.3	4.1	Abbas-Abadi et al. (2014)
Semi-batch (semi-flow)	PS	400	7	1 atm	—	4	90	6	Kyong et al. (2002)
Fluidized bed (multiphase)	HDPE	500	—	—	60	5	85	10	Luo et al. (2000)
Fluidized bed (multiphase)	HDPE	650	—	—	23	0	68.5	31.5	Mastral et al. (2001)
Fluidized bed (multiphase)	LDPE	600	—	1 atm	—	0	51.0	24.2	Williams and Williams, (1998)
Pressurized batch	PS	425	10	0.31–1.6 MPa	60	0.5	97	2.50	Onwudili et al. (2009)
Horizontal steel	PP	300	20	—	30	1.34	69.82	28.84	Ahmad et al. (2014)
	LDPE	500	6	1 atm	—	0.16	80.40	19.43	Fakhrhoseini and Dastanian, (2013)
Pressurized batch	LDPE	425	10	0.8–4.3 MPa	60	0.5	89.53	10	Onwudili et al. (2009)
Vacuum batch	PVC	520	10	2 kPa	—	28.13	12.79	0.34	Miranda et al. (1998)
Horizontal steel	HDPE	350	20	—	30	1.88	80.88	17.24	Ahmad et al. (2014)
	PET	500	6	1 atm	—	8.98	38.89	52.13	Fakhrhoseini and Dastanian, (2013)
Fixed bed (packed)	PET	500	10	—	—	—	23.1	76.9	Cepeliogullar et al. (2013)
Fixed bed (packed)	PVC	500	10	—	—	0	12.3	87.7	Mastral et al. (2001)
Fixed bed (packed)	LDPE	500	10	—	20	0	95	5	Bagri and Williams, (2001)

In thermal degradation, the ideal temperature in plastic pyrolysis for maximizing liquid oil production is between 500 and 550°C as shown in Table 11. Nevertheless, the utilization of the catalyst in plastic waste pyrolysis allowed the optimal temperature to be decreased to 450°C, resulting in a significant increase in liquid yield production. Among the plastics, polystyrene (PS) is the best plastic for the pyrolysis process and produced 97 wt% of liquid oil without any catalyst compulsions (Onwudili et al., 2009). In terms of polyolefin plastic types in thermal pyrolysis, PP provided the lowest yield of liquid oil (82.12 wt%) and LDPE provided the highest (93.1 wt%). However, product optimization of 90 wt% or above is possible by using the appropriate catalysts and performing experiments at the right operating parameters.

The preparation of useful materials in tribology is also an interesting application of plastic waste recycling (Iqbal et al., 2020; Iqbal et al., 2022). This is another alternative to disposing of plastic waste and recycles it to develop lubricating oil for tribological applications. Recently, Hackler et al. (2021) compared the tribological performance of synthetic lubricants derived from HDPE, LLDPE, and bubble wrap with industrial-grade oils. Their findings suggest that the lubricants derived from the waste plastics outperformed the traditional mineral oil with a 43% improvement in wear volume when compared to Group III minerals. Furthermore, Sikdar et al. (2020) studied the frictional behavior of pyrolyzed oils derived from waste plastics, and their results indicate that these pyrolyzed plastic waste oils exhibit similar frictional behavior when compared to bio-based lubricants. Moreover, the waxes obtained during polyolefin plastics (PP and PE) fast pyrolysis and oil produced during tire pyrolysis together can be co-fed with the industrial current stream units. It is an opportunity for conventional refineries to operate as a waste refineries by co-feeding these feeds alternatively and adjusting the fuel characteristics and raw materials produced, to be tailored to commercial objectives within the oil economy framework (Palos et al., 2021).

Considering the Sustainable Development Goals (SDGs), lubricants derived from pyrolysis and gasification of plastic waste not only have the potential to reduce plastic pollution but also the potential to replace industrial-grade oils for tribological applications.

## 13 Conclusion

This study gives a comprehensive overview of gasification and plastic pyrolysis for each classification, as well as a discussion of the most important influencing aspects for optimizing H_2_ production and liquid oil yield. In contrast to conventional combustion (incineration), one of the key contentions for gasification and pyrolysis is to enhance ecological performance and the possibility for ameliorating emission control. In the literature studies, most researchers have preferred pyrolysis process over gasification because it has the greatest potential for converting most of the waste plastics energy into useful char, gas, and liquid oil. The fundamental obstacle of gasification of plastic waste is the formation of tar, which leads to major operational challenges, thus reducing the gas yield and influencing the total process productivity. The pyrolysis process also has drawbacks, such as a more complex product stream and the inability to directly vent product gases due to high concentrations of CO. The composition and variable quality of the feed is a considerable challenge for all plastic conversion processes. The long-term viability of these processes are indisputable because by using these valorization routes, the management of waste becomes highly systematic, with less landfill space required, lower cost, and less pollution. As a final conclusion, the ideal way to encounter plastic pollution is to recycle plastic waste either by gasification or pyrolysis.

## Abbreviations

MSW, municipal solid waste; PE, polyethylene; HDPE, high-density polyethylene; FBR, fluidized bed reactor; CBF, circulating fluidized bed; BFB, bubbling fluidized bed; DFB, dual fluidized bed; CSBR, conical spouted bed reactor; PP, polypropylene; LDPE, low-density polyethylene; MJ, mega joules; EDAX, energy-dispersive X-ray spectroscopy; WGS, water–gas shift; ER, equivalence ratio; PET, polyethylene terephthalate; PVC, polyvinyl chloride; PS, polystyrene; PETRA, the PET Resin Association; EPA, Environmental Protection Agency; GC-MS, gas chromatography–mass spectroscopy; HIP, high-impact polystyrene; RPM, revolutions per minute; TG, thermogravimetry analysis; DTG, derivative thermogravimetry analysis; SEM, scanning electron microscope; FFC, fluid catalytic cracking; SDG, sustainable development goal.
